# Substituent Effects in the Photophysical and Electrochemical Properties of Meso-Tetraphenylporphyrin Derivatives

**DOI:** 10.3390/molecules29153689

**Published:** 2024-08-04

**Authors:** Alexandra Cruz Millheim, Enric Ponzano, Albert Moyano

**Affiliations:** Section of Organic Chemistry, Department of Inorganic and Organic Chemistry, Faculty of Chemistry, University of Barcelona, C. de Martí i Franquès 1-11, 08028 Barcelona, Spain; acruzmillheim@gmail.com (A.C.M.); eponzasa13@alumnes.ub.edu (E.P.)

**Keywords:** photosensitizers, photoredox catalysis, porphyrins, redox potential, substituent effects

## Abstract

Porphyrins were identified some years ago as a promising, easily accessible, and tunable class of organic photoredox catalysts, but a systematic study on the effect of the electronic nature and of the position of the substituents on both the ground-state and the excited-state redox potentials of these compounds is still lacking. We prepared a set of known functionalized porphyrin derivatives containing different substituents either in one of the meso positions or at a β-pyrrole carbon, and we determined their ground- and (singlet) excited-state redox potentials. We found that while the estimated singlet excited-state energies are essentially unaffected by the introduction of substituents, the redox potentials (both in the ground- and in the singlet excited-state) depend on the electron-withdrawing or electron-donating nature of the substituents. Thus, the presence of groups with electron-withdrawing resonance effects results in an enhancement of the reduction facility of the photocatalyst, both in the ground and in the excited state. We next prepared a second set of four previously unknown meso-substituted porphyrins, having a benzoyl group at different positions. The reduction facility of the porphyrin increases with the proximity of the substituent to the porphine core, reaching a maximum when the benzoyl substituent is introduced at a meso position.

## 1. Introduction

Visible-light photocatalysis has become an important and well-established synthetic method for the construction of complex molecular architectures, thanks to the renaissance of the field brought about by the influential work of MacMillan [1], Yoon [2], and Stephenson [3], among others [4,5,6,7,8,9]. Visible-light photoreactions are made possible by the existence of photocatalysts (PCs), compounds that are promoted to their electronic excited states through irradiation with light; the resulting highly energetic species can exchange energy with the surrounding molecules that do not absorb visible radiation (photosensitization) [7] or engage in single-electron transfer (SET), leading to photoredox catalysis [4,5,8,9]. Most molecular PCs are inorganic or organometallic complexes of Ru(II), Ir(III), or Cu(I), but metal-free organic dyes have emerged as low-cost and green alternatives [10]. The rational design of new photocatalysts with designed and improved photocatalytic properties, which, at the same time, circumvents the necessity of using scarce and toxic transition metals, will predictably play a key role in photocatalysis in the next few years.

**Scheme 1:**
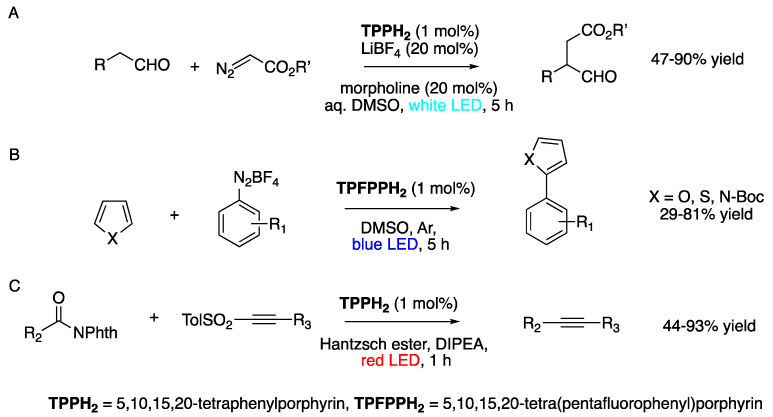
Some applications of porphyrins as photoredox catalysts. A: α-alkylation of aldehydes with diazo esters [15]. B: arylation of heterocycles [16]. C: desulfonilative alkylation of alkynyl sulfones [17].

Notwithstanding the widespread use of porphyrins as photosensitizers for singlet oxygen generation [11,12,13,14], this important class of organic dyes had been not considered a source of photoredox catalysts until the seminal report by Zawada, Kadish, and Gryko [15], published in 2016. Subsequently, other photocatalytic applications of porphyrins have emerged [16,17], but the number of porphyrin-based photoredox catalysts is still very limited, particularly when only free-base porphyrins are considered (Scheme 1).

In the context of our research on the use of porphyrins as macromolecular catalysts [18,19] and as switchable organocatalysts [20,21], we have been recently interested in the photocatalytic activity of functionalized porphyrins [22]. While several studies have been performed concerning the redox electrochemical behavior of metalloporphyrins [23,24], much less quantitative data are available on the photophysical and electrochemical properties of free-base porphyrins [15,16,25,26], and a systematic study on the effect of the electronic nature and of the position of the substituents on both the ground-state and the excited-state redox potentials of these compounds is still lacking. This is surprising, especially taking into account that, as already recognized by Gryko et al. [15], porphyrins are accessible by a variety of synthetic methods [27,28,29] and that their photophysical and electrochemical properties should be readily tuned by the introduction of adequate substituents in the different positions of the porphyrin core.

**Figure 1:**
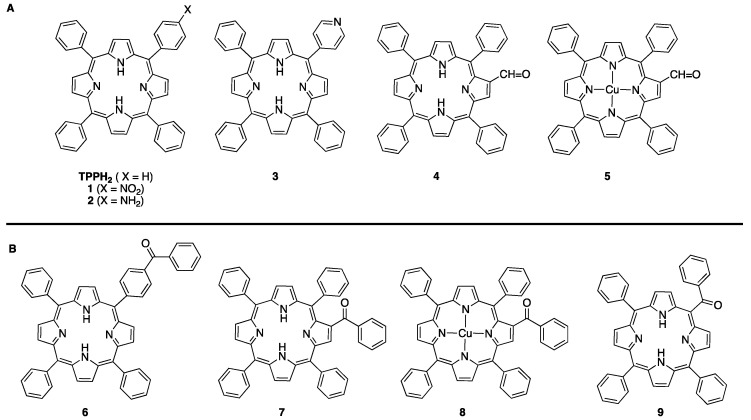
Porphyrin derivatives studied in this work: (**A**): previously known compounds. (**B**): new benzoyl-porphyrin derivatives.

We decided, therefore, to prepare a set of known functionalized porphyrins (**1**–**5**) containing different substituents either in one of the meso-positions (**1**–**3**) or at a β-pyrrole carbon (**4**,**5**), and to determine their ground- and (singlet) excited-state redox potentials (Figure 1A). This first survey allowed us to conclude that the introduction of electron-withdrawing groups, particularly at the β-pyrrole position, increases the facility of the monoelectronic reduction of the porphyrin both in the ground state and in the singlet excited state. In light of these results, we prepared a second set of four previously unknown **TPPH_2_** benzoyl derivatives (**6**–**9**), (Figure 1B), which confirmed the validity of our conclusions regarding the facility of reduction, and that allowed us to ascertain the influence of the position of the substituent.

## 2. Results and Discussion

We proceeded in the first place to record the cyclic voltammograms (CVs) of the known porphyrins **TPPH_2_** and **1**–**5**, in order to evaluate their ground-state redox potentials. After checking the solubility of our porphyrin set in various solvents, we selected dichloromethane (DCM) as the solvent, and tetrabutylammonium perchlorate (TBAP, 0.1 M) as the supporting electrolyte. The results obtained are gathered in Table 1 (entries 1–6), after correction of the obtained values (Ag/AgCl reference electrode) to the standard calomel electrode (SCE, +0.05 V correction). The CVs of **TPPH_2_** (in DMSO) [15] and of 5-(*p*-nitrophenyl)-10,15,20-triphenylporphyrin **1** [26] have already been reported, and we were pleased to find that our results were essentially the same.

Subsequently, the UV–Vis and the fluorescence spectra of the porphyrins were measured in DCM, to estimate the singlet excited-state energies (E^S1^_0,0_). We determined the wavelength at the intersection between the normalized less energetic UV–Vis absorption (the most red-shifted Q band) and the more energetic emission band (Q_0,0_) [7,30]. Contrary to what happens with the redox potentials, in the case of free-base porphyrins **1**–**3**, this wavelength does not depend on the presence and nature of the substituents and was essentially the same for all of them (648 ± 1 nm), so that a common value of 1.91 V can be used for the energy of the first singlet excited state for compounds **1**–**3**. A somewhat smaller value (1.86 V) was found for the β-formyl substituted porphyrin **4**.

In the case of Cu complex **5**, however, the intersection wavelength takes a rather different value (620 ± 1 nm), so that for this metalloporphyrin, the estimated singlet excited-state energy is 2.00 V. It is worth noting that this value is in good agreement with the reported zero–zero excitation energy of 2.04 V of **ZnTPP** [15].

Combining the estimated singlet excited-state energies (E^S1^_0,0_) with the ground-state redox potentials extracted for the first SET processes and making use of the commonly used approximations [6], we obtain the excited-state potentials. The combined data set is summarized in Table 2 (entries 1–6).

An inspection of the results gathered in Table 2 shows that the introduction of substituents having non-bonding electron pairs in a nitrogen atom (compounds **2** and **3**, entries 3 and 4) increases the facility of oxidation of the excited state (column 6), while the presence of a nitro (**1**, entry 2) or a β-formyl group (compound **4**, entry 5), which are electron-withdrawing substituents with less oxidable oxygen-centered lone electron pairs, has a small effect in the opposite direction. As expected from the dianionic character of the porphyrin core, Cu-complex **5** (entry 6, column 6) is more easily oxidized in the excited state than the free-base porphyrin **4** (entry 5, column 6). The stronger substituent effects are seen, however, when one compares the relative facility for the reduction in the excited state (column 5), which can be directly related to the electron-donating or electron-accepting nature of the substituents. Thus, the presence of a *p*-amino group in one of the meso phenyls (compound **2**) practically does not change the reduction potential with respect to **TPPH_2_** (+0.69 V vs. +0.70 V), probably due to the compensation between the electron-retrieving inductive effect and the electron-donating resonance effect of this group. The presence of a *p*-nitro (compound **1**) or of a β-formyl group (compound **4**) substantially increases the facility of reduction (by +0.15 V and by +0.21 V, respectively). When Cu(II) complex **5** is examined (entry 6 in Table 2), the effect of the formyl substituent is somewhat diminished (+0.23 V with respect to **TPPH_2_**). The replacement of a meso-phenyl group by a 4′-pyridyl moiety (compound **3**) brings about a smaller increase (+0.07 V) in the reduction potential of the excited photocatalyst. We concluded, therefore, that the introduction of a carbonyl substituent strongly increases the facility of reduction in the excited state of the photocatalyst, tuning its photoredox properties toward a reductive quenching cycle.

In the case of **TPPH_2_**, the approximate triplet excited-state redox potentials can be readily evaluated, since a −0.50 V energy gap between the singlet and the triplet has been experimentally determined [31,32]. Although the singlet–triplet difference energy is not known for the other porphyrins, given that the singlet energy value appears to be independent of the substituents, we can assume a similar energy gap for the free-base porphyrins **1**–**4**, so that the corresponding triplet excited-state potentials can also be readily estimated. In any case, the observed substituent effects will hold also for the triplet excited states.

In light of these results, we decided to perform a similar study in a set of previously unknown meso-substituted porphyrin derivatives (**6**–**9**, Figure 1B), having a benzoyl group at different positions. In this way, we wanted to confirm that the presence of carbonyl substituent increased the facility of reduction in the excited state, and to explore the effect of the position of the benzoyl moiety. The change of the formyl to the benzoyl substituent was motivated by the diminished chemical reactivity of the latter, in the first place, and by the possibility of observing the photocatalytic hydrogen atom transfer (photoHAT) activity of diaryl ketones [33], in the second place.

**Scheme 2:**
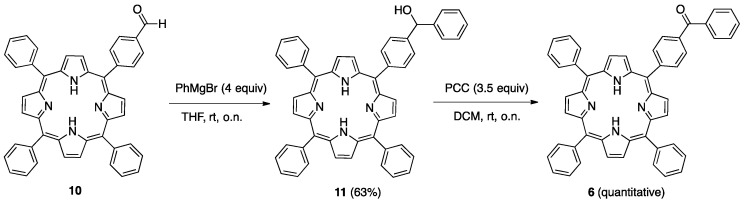
Synthesis of 5-(4′-benzoyl)-10,15,20-triphenylporphyrin **6**.

The 5-(4′-benzoyl)-10,15,20-triphenylporphyrin **6** was easily obtained in two steps from the known [11] 5-(4′-formyl) derivative **10** (Scheme 2). Treatment of this compound with an excess of phenylmagnesium bromide in anhydrous THF produced the benzhydryl alcohol **11**, which was subsequently oxidized by pyridinium chlorochromate to provide **6** in 63% overall yield, after chromatographic purification.

**Scheme 3:**
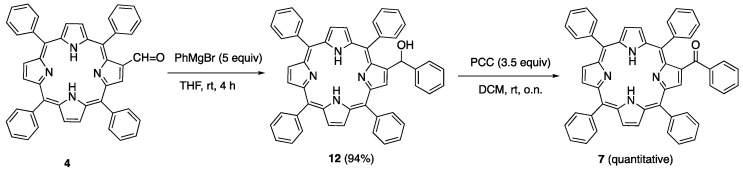
Synthesis of benzoyl-porphyrin derivative **7**.

**Scheme 4:**
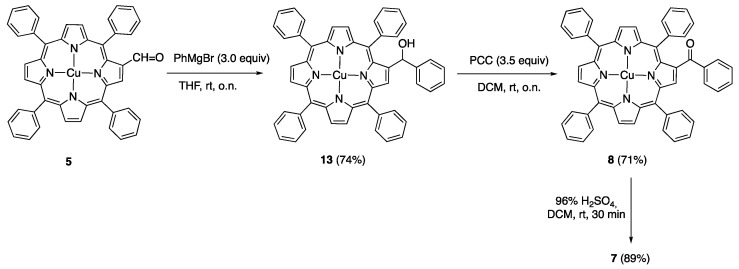
Synthesis of porphyrin copper complex **8** and acid-promoted demetallation to **7**.

On the other hand, phenyl(5,10,15,20-tetraphenylporphyrin-7-yl)methanone **7** could be accessed either from the β-formyl derivative **4** [22,34] (Scheme 3) or from the corresponding Cu-complex **5** [22,35] (Scheme 4).

As in the case of **6** above, the β-benzoyl derivative **7** was synthesized through addition of the phenyl Grignard reagent (in excess, to compensate for the loss of phenylmagnesium bromide by deprotonation of the porphyrin core) to the β-formyl substituted porphyrin **4**, and the resulting alcohol **12** (obtained in 94% yield) was oxidized quantitatively with PCC (Scheme 3).

Starting from Cu complex **5**, the same reaction sequence afforded Cu complex **8**, albeit in a somewhat lower overall yield. Acid-catalyzed demetallation of **8** to give **7** took place with good yield (Scheme 4).

**Scheme 5:**
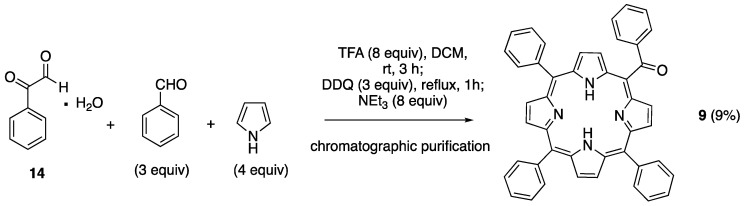
Synthesis of 5-(benzoyl)-10,15,20-triphenylporphyrin **9**.

Finally, 5-(benzoyl)-10,15,20-triphenylporphyrin **9** was obtained by the mixed condensation of phenylglyoxal monohydrate **14** with benzaldehyde and pyrrole (Scheme 5), following the conditions previously described by Lindsey for the preparation of 5-(benzoyl)-10,15,20-tris(*p*-tolyl)porphyrin in 13% yield [29]. In a single step, after chromatographic purification, pure **9** was isolated in 9% yield.

We recorded next the CVs of these benzoyl-substituted porphyrins, in DCM solution. The results are summarized in Table 1 above (entries 7–10).

We were pleased to find that, according to our prediction, the introduction of an electron-withdrawing benzoyl group resulted in a greater facility of reduction of the neutral species with respect to the unsubstituted meso-tetraphenylporphyrin (compare, for instance, the values for the first monoelectronic reduction in column 2 of Table 1), and at the same time, making more difficult the first monoelectronic oxidation (column 3 of Table 1). It should also be noted that these effects increased with the proximity of the benzoyl group to the porphine core, so that 5-(benzoyl)-10,15,20-triphenylporphyrin **9** (entry 10 in Table 1) exhibits a less negative value for the monoelectronic reduction potential of the neutral molecule (−0.97 V vs. −1.21 V for **TPPH_2_**) and a more positive value for the reduction of the radical cation (+1.32 V vs. +1.09 V for **TPPH_2_**).

The photophysical properties of porphyrins **6**–**9**, derived from their UV–Vis and fluorescence spectra, are summarized in Table 3. As can be seen, the benzoyl-substituted porphyrins **6** and **9** have the same normalized intersection wavelength (648 nm) and, therefore, the same estimated singlet excited-state energy (1.91 V) as the previously studied set of free-base porphyrins (**TPPH_2_**, **1**–**4**), together with very low values for the wavelength difference between the maxima of the absorption and of the emission bands (3–6 nm). The pyrrolyl-substituted porphyrin **7**, on the other hand, has a slightly higher intersection wavelength (657 nm), showing therefore a correspondingly lower (by 0.02 V) singlet excited-state energy. Finally, Cu(II) complex **8** behaved, as expected, rather differently than the other members of the set: it presents a low intersection wavelength (618 nm), a large wavelength difference between the absorption and the emission maxima (63 nm), and a high singlet excited-state energy (2.00 V). This value is the same observed for β-formyl Cu-complex **5**, showing again that it is independent of the nature of the substituents on the porphyrin.

We measured also the fluorescence quantum yields for these compounds (column 5 in Table 3). The free-base porphyrins **6**, **7**, and **9** presented values only slightly inferior to that of **TPPH_2_** (which was used as the reference compound), because of the extended π-system. The well-known “heavy atom effect” [36] of porphyrin Ni(II) and Cu(II) complexes was readily apparent in complex **8**, with a very low fluorescence quantum yield.

With the measured values of the singlet excited states in our hands, we could finally evaluate both the ground- and the excited-state redox potentials of benzoyl-substituted porphyrins **6**–**9**, which are shown in Table 2 above (entries 7–10).

By inspection of the values gathered in Table 2, we can see that the introduction of a benzoyl group structure increases the facility of monoelectronic reduction (relative to that of **TPPH_2_**) both in the ground state (column 2 in Table 2) and in the excited state (column 4 in Table 2). At the same time, the monoelectronic oxidation (columns 3 and 5 in the Table 2) shows the opposite behavior. As happened when we compared the free-base formyl-substituted porphyrin **4** (entry 5 in Table 2) with Cu-complex **5** (entry 6 in Table 2), the metallated β-benzoyl derivative **8** (entry 9 in Table 2) is somewhat less easily reduced, in the ground state, than the free-base **7** (entry 8 in Table 2). Thus, the presence of π-conjugated, electron-withdrawing substituents, such as formyl or benzoyl, enhances the reduction facility of meso-substituted porphyrins. Moreover, we can see that this effect depends on the position of the benzoyl substituent. This becomes readily apparent when we compare the reduction facility in the excited state (column 4 in Table 2). The reduction potential becomes more positive along this column. Thus, the effect of a benzoyl group on the *p*-position of a meso-phenyl moiety (porphyrin **6**, entry 7) is smaller than in a β-pyrrole position (porphyrin **7**, entry 8), which, in turn, is less than in a meso-position (porphyrin **9**, entry 10). This effect is somewhat diminished in the Cu complex **8** (entry 9), which nevertheless is more easily reduced, both in the ground and in the (singlet) excited state, than **TPPH_2_** (entry 1). The facility of reduction increases therefore with the proximity of the electron-accepting substituent to the porphine core. Consequently, the most easily reduced compound in the series is the meso-benzoyl derivative **9**. This compound exhibits a half-wave reduction potential in the excited state of +0.94 V ([*PC/PC^.−^]), and should therefore behave as a good photooxidant in a reductive quenching cycle.

## 3. Materials and Methods

### 3.1. General Methods

Commercially available reagents, catalysts, and solvents were used as received from the supplier. Dichloromethane for porphyrin synthesis was distilled from CaH_2_ prior to use, and THF was dried by distillation from LiAlH_4_. Deuterated solvents were supplied by Merck Life Science.

Thin-layer chromatography was carried out on silica gel plates Merck 60 F_254_, and compounds were visualized by irradiation with UV light and/or chemical developers (KMnO_4_, *p*-anisaldehyde, and phosphomolybdic acid). Chromatographic purifications were performed under pressurized air in a column with silica gel Merck 60 (particle size: 0.040–0.063 mm, Merck Life Science S.L.U., 28006-Madrid, Spain) as stationary phase and solvent mixtures (hexane, ethyl acetate, dichloromethane, and methanol) as eluents.

NMR spectra (^1^H, 400 MHz; ^13^C, 101 MHz) were recorded with a Varian Mercury 400 spectrometer (Agilent Technologies, Santa Clara, CA, USA). Chemical shifts (δ) are given in ppm relative to the peak of tetramethylsilane (δ = 0.00 ppm) and coupling constants (*J*) are provided in Hz. The spectra were recorded at room temperature. Data are reported as follows: s, singlet; d, doublet; t, triplet; q, quartet; m, multiplet; br, broad signal. IR spectra were obtained with a Nicolet 6700 FTIR instrument (Thermo Fisher Scientific, Waltham, MA, USA), using ATR techniques.

### 3.2. Photophysical Methods

UV–Vis spectra were recorded on a double-beam Cary 500-scan spectrophotometer (Varian); cuvettes (quartz QS Suprasil, Hellma, Hellma GmbH & Co. KG, Mülheim, Germany) of 1 cm were used for measuring the absorption spectra. All spectra were carried out in DCM freshly filtered over basic Al_2_O_3_, to eliminate possible traces of hydrochloric acid. Fluorescence spectra measurements were carried out in a PTI Felix GX spectrofluorometer. All spectra were recorded in an open window of 1.5 mm. The optical path of the cell was 1 cm.

The relative method for the calculation of the fluorescence quantum yield was used, taking **TPPH_2_** [(ϕ_f_) = 0.11] as the reference compound. The reference and the new substance were irradiated at the same wavelength (420 nm), at the same concentration (10^−6^ M) in DCM solution, and with the same slit distance. After evaluation of the areas (*I*, *I_R_*) by integration of the intensity curve of the emission spectra of the porphyrin under study and of the reference compound, the fluorescence quantum yields were given by the formula

$$\phi =\phi_{R}•\;\frac{I}{I_{R}}•\frac{Abs_{R}}{Abs}$$

where $\phi_{R}$ = 0.11, and *Abs_R_* and *Abs* correspond to the maximum absorbance of the reference compound and of the studied porphyrin at the irradiation wavelength, respectively.

### 3.3. Cyclic Voltammetry

Cyclic voltammetry measurements were carried out with a computer-controlled potentiostat Model Epsilon EClipse (BASi). The setup comprised an undivided cell, a glassy carbon working electrode (3 mm diameter), a platinum wire counter electrode, and a saturated Ag/AgCl reference electrode. Each cyclic voltammetry was acquired at a sweep rate of 100 mV/s. The examined solvents were initially DCM, DMSO, DMF, and MeCN using TBAP (0.1 M) as the supporting electrolyte in 50 mL of solution. The best results for the whole set of porphyrins, due to solubility reasons, were obtained for DCM. For the CVs, the reaction mixtures were previously purged under argon to avoid the presence of oxygen during the measurements. To perform the experiments and detect the presence of possible catalytic current, 5 mM stock solutions of the synthesized porphyrins were prepared

### 3.4. Synthetic Procedures and Product Characterization

**5-(4′-Benzoyl)-10,15,20-triphenylporphyrin (6)**

A 1 M THF solution of phenylmagnesium bromide (0.62 mL, 0.62 mmol, 4 equiv.) was added dropwise to a flask containing a cold (−78 °C, dry ice-acetone bath) solution of the known aldehyde **10** [22] (100 g, 0.16 mmol) in anhydrous THF (30 mL) under an argon atmosphere. When the addition was finished, the reaction mixture was allowed to warm up to room temperature and stirred for 4 h, at which time, it was carefully quenched with aqueous sat. NH_4_Cl (2 mL). The solution was distilled under vacuum, evaporating most of the THF. In a separatory funnel, the organic phase was separated, and the aqueous phase was washed with ethyl acetate (3 × 10 mL). The combined organic phases were washed with brine (5 mL) and dried over anhydrous MgSO_4_. The solvent was evaporated under vacuum. The product was then purified via column chromatography on silica gel (1:1 hexane:DCM) to afford alcohol **11** (77 mg, 63% yield).

**^1^H NMR** (400 MHz, CDCl_3_) δ 8.84 (s, 8H), 8.26–8.16 (m, 8H), 7.81–7.70 (m, 11H), 7.71–7.64 (m, 2H), 7.55–7.47 (m, 2H), 7.43–7.38 (m, 1H), 6.21 (s, 1H), 3.60 (s, 1H OH), −2.78 (s, 2H NH) ppm. **^13^C NMR** (101 MHz, CDCl_3_) δ 144.1, 143.3, 142.3, 141.6, 134.9, 134.7, 128.9, 128.1, 127.9, 127.0, 126.8, 125.1, 120.3, 119.9, 76.6 ppm.

Without further characterization, the porphyrin alcohol **11** (166 mg, 0.23 mmol) was directly diluted with DCM (20 mL) and then slowly added to a well-stirred mixture of pyridinium chlorochromate (173 mg, 0.80 mmol, 3.5 eq) and silica gel (0.4 g) in DCM (10 mL). After the addition, the reaction was monitored by TLC. Once completion of the reaction was observed (12 h stirring at rt), the reaction mixture was filtered through a short pad of silica gel and was washed several times with DCM, to afford the desired ketone **6** (163 mg, quantitative yield).

**^1^H NMR** (400 MHz, CDCl_3_) δ 8.95–8.85 (m, 8H), 8.38 (ddd, J = 8.4, 4.3, 2.0 Hz, 2H), 8.28–8.21 (m, 8H), 8.12 (ddt, J = 7.0, 3.3, 1.5 Hz, 2H), 7.85–7.74 (m, 9H), 7.70 (d, J = 7.1 Hz, 1H), 7.64 (td, J = 7.6, 7.0, 1.8 Hz, 2H), −2.72 (bs, 1H NH), −2.74 (bs, 1H NH) ppm.

**^13^C NMR** (101 MHz, CDCl_3_) δ 197.0, 146.8, 142.2, 137.9, 136.9, 134.7, 134.7, 132.8, 130.4, 128.7, 128.6, 127.9, 126.9, 120.8, 120.6, 118.6 ppm.

**HRMS (ESI)** *m*/*z* calculated for C_51_H_35_N_4_O [M+H]^+^, 719.2805; found, 719.2803. *m*/*z* calculated for C_51_H_36_N_4_O [M+2H]^2+^, 360.1439; found, 360.1448.

**UV–Vis** [DCM, λ_max_ (logε), C = 7.2 × 10^−5^ M]: 419 (4.78), 515 (3.44), 550 (3.05), 590 (2.83), 645 (2.66).

**FTIR** (solid,$\;\bar{\nu \;}\left({\mathrm{cm}}^{-1}\right))$ = 1653 (CO)

**Phenyl(5,10,15,20-tetraphenylporphyrin-7-yl)methanone (7)**

A 1M THF solution of phenylmagnesium bromide (1.4 mL, 1.4 mmol, 5 equiv.) was added dropwise to a flask containing a cold (−78 °C., dry-ice/acetone bath) solution of the known [22,34] 2-formyl-5,10,15,20-tetraphenylporphyrin **4** (183 mg, 0.28 mmol) in anhydrous THF (60 mL) under an argon atmosphere. The reaction was allowed to warm up to room temperature and stirred for 4 h, at which time, it was carefully quenched with 5 mL of aq. sat. NH_4_Cl solution. The organic phase was separated, the aqueous phase was extracted with ethyl acetate (3 × 15 mL), and the combined organic phases were washed with brine (10 mL) and dried over anhydrous MgSO_4_. After rotary evaporation, the crude product was then purified via column chromatography in silica gel, eluting with 1:1 hexane:DCM, to afford the alcohol **12** (192 mg, 94% yield).

**^1^H NMR** (400 MHz, CDCl_3_) δ 9.05 (s, 1H), 8.86 (dq, J = 12.1, 4.8 Hz, 4H), 8.74 (d, J = 4.8 Hz, 1H), 8.52 (d, J = 4.8 Hz, 1H), 8.32–8.11 (m, 7H), 7.82–7.30 (m, 13H), 7.20–7.12 (m, 1H), 7.09 (tt, J = 8.2, 6.7, 1.3 Hz, 2H), 6.97–6.92 (m, 2H), 6.21 (s, 1H), 3.09 (s, 1H OH), −2.65 (s, 2H NH) ppm. **^13^C NMR** (101 MHz, CDCl_3_) δ 144.0, 142.6, 142.3, 142.0, 141.6, 134.8, 134.7, 134.3, 133.7, 132.3, 128.4, 128.0, 127.9 (x2), 127.7, 126.9 (x3), 126.8 (x2), 120.6, 120.4, 120.1, 119.8, 72.0 ppm.

Without further characterization, porphyrin alcohol **12** (133 mg, 0.18 mmol) was diluted with DCM (20 mL) and then slowly added to a stirred mixture of pyridinium chlorochromate (139 mg, 0.65 mmol, 3.5 equiv.) and silica gel (0.10 g) in DCM (15 mL). After the addition, the reaction was monitored by TLC. Once completion of the reaction was observed (12 h stirring at rt), the reaction mixture was filtered through a short pad of silica gel and was washed several times with DCM, to afford the desired ketone **7** (131 mg, quantitative yield).

**^1^H NMR** (400 MHz, CDCl_3_) δ 9.01 (s, 1H), 8.94 (q, J = 4.9 Hz, 2H), 8.87–8.76 (m, 4H), 8.30–8.19 (m, 6H), 7.87–7.69 (m, 11H), 7.50 (tt, J = 7.5, 1.2 Hz, 1H), 7.42–7.35 (m, 3H), 7.28 (t, J = 7.9 Hz, 2H), 7.16–7.06 (m, 2H), −2.62 (s, 2H) ppm.

**^13^C NMR** (101 MHz, CDCl_3_) δ 195.0, 142.2, 141.9, 141.9, 141.0, 138.6, 136.7, 134.8, 134.8, 132.7, 129.5, 128.1, 128.0, 127.7, 127.0, 126.9, 126.9, 126.6, 121.2, 121.1, 120.6, 120.5 ppm.

**HRMS (ESI)** *m*/*z* calculated for C_51_H_35_N_4_O [M+H]^+^, 719.2805; found, 719.2801. *m*/*z* calculated for C_51_H_36_N_4_O [M+2H]^2+^, 360.1439; found, 360.1446.

**UV–Vis** [DCM, λ_max_ (logε), C = 7.2 × 10^−6^ M]: 422 (4.92), 519 (3.50), 555 (3.02), 596 (2.92), 652 (2.96).

**FTIR** (solid,$\;\bar{\nu \;}\left({\mathrm{cm}}^{-1}\right))$ = 1655 (CO)

**Phenyl(5,10,15,20-tetraphenylporphyrin-7-yl)methanone copper(II) complex (8)**

A 1M THF solution of phenylmagnesium bromide (1.9 mL, 1.9 mmol, 3.0 equiv.) was added dropwise to a round-bottom flask containing a cold (−78 °C, dry-ice/acetone bath) solution of the known [22,35] copper(II) 2-formyl-5,10,15,20-tetraphenylporphyrin **5** (455 mg, 0.65 mmol) in anhydrous THF (60 mL) under an argon atmosphere. The flask was allowed to warm up to room temperature and stirred overnight. Then, the reaction mixture was carefully quenched with aq. sat. NH_4_Cl solution (5 mL). The aqueous phase was extracted with ethyl acetate (3x10 mL), and the combined organic phase was washed with brine (10 mL), dried over anhydrous MgSO_4,_ and concentrated under vacuum. The crude product was then purified by column chromatography (silica gel, 1:1 hexane:DCM) to afford 372 mg (74% yield) of the desired alcohol **13**, which was not further characterized.

A solution of porphyrin alcohol **13** (372 mg, 0.47 mmol) in DCM (60 mL) was slowly added to a well-stirred suspension of pyridinium chlorochromate (361 mg, 1.7 mmol, 3.6 equiv.) and silica gel (0.4 g) in DCM (10 mL). After the addition, the reaction was monitored by TLC. Once completion of the reaction was observed (12 h stirring at rt), the reaction mixture was filtered through a short pad of silica gel and was washed several times with DCM, to afford the desired ketone **8** (264 mg, 71% yield).

**HRMS (ESI)** *m*/*z* calculated for C_51_H_33_N_4_OCu [M+H]^+^, 780.1945; found, 780.1944. *m*/*z* calculated for C_102_H_65_N_8_O_2_Cu_2_ [2M+H]^+^, 1559.3817; found, 1559.3824.

**UV–Vis** [DCM, λ_max_ (logε), C = 7.4 × 10^−5^ M]: 420 (4.75), 545 (3.48), 587 (2.99).

**FTIR** (solid,$\;\bar{\nu \;}\left({\mathrm{cm}}^{-1}\right))$ = 1653 (CO)

The identity of **8** was further established by acid-promoted demetallation to **7**: In a 50 mL round-bottom flask, equipped with magnetic stirring, Cu(II) porphyrin complex **8** (26.3 mg, 0.034 mmol) was dissolved in 5 mL of DCM. Concentrated H_2_SO_4_ (1 mL, 98%) was added, and the green mixture was vigorously stirred for 30 min. Then, the two-phase mixture was carefully poured into a cold (0ºC) aqueous NaOH solution (1 g in 20 mL), transferred into a separatory funnel, and shaken until no green color was observed. The aqueous phase was extracted with DCM (3 × 10 mL), and the combined organic layers were washed with an aqueous saturated solution of NaHCO_3_ (2 × 10 mL), dried over MgSO_4_, and the solvent was evaporated under reduced pressure. Finally, the obtained crude product was purified via flash chromatography through Et_3_N-pretreated silica gel (2.5% *v*/*v* NEt_3_) using DCM/AcOEt (1/1) as eluent to obtain the free-base porphyrin **7** (21.5 mg), in 89% yield.

**5-(Benzoyl)-10,15,20-triphenylporphyrin (9)**

A 1 L round-bottom reaction flask was successively charged with a DCM solution (800 mL) of phenylglyoxal monohydrate **14** (0.302 g, 2.0 mmol), and was purged with argon for 15 min. Then, freshly distilled pyrrole (0.56 mL, 8.0 mmol) and benzaldehyde (0.64 mL, 6.0 mmol) were added. The resulting mixture was stirred for 5 min and trifluoroacetic acid (1.22 mL, 16 mmol) was added dropwise. At this point, a change in color was observed. The flask was covered with aluminum foil to protect the reaction from light. After 3 h of stirring at rt, DDQ (1.36 g, 6.0 mmol) was added, and the resulting solution was stirred under reflux for 1 h. After cooling to rt, triethylamine (2.2 mL, 16 mmol) was added dropwise. The solvents were evaporated in vacuo, and the resulting residue was submitted to column chromatographic purification (silica gel, DCM gradient with ethyl acetate). A middle-polarity fraction containing a mixture of monosubstituted and disubstituted porphyrins was separated. A second chromatographic purification (silica gel, DCM/hexanes 1:1) furnished the desired porphyrin **9** (115 mg, 9% yield).

**^1^H NMR** (400 MHz, CDCl_3_) δ 9.03 (d, J = 4.8 Hz, 2H), 8.92–8.84 (m, 6H), 8.27–8.16 (m, 6H), 7.96 (d, J = 7.6 Hz, 2H), 7.85–7.72 (m, 9H), 7.59 (tt, J = 7.6, 1.3 Hz, 1H), 7.41 (ddd, J = 9.1, 7.5, 1.5 Hz, 2H), −2.69 (s, 2H) ppm.

**^13^C NMR** (101 MHz, CDCl_3_) δ 199.5, 142.1, 142.0, 141.8, 134.7, 134.6, 133.6, 131.5, 128.6, 128.1, 128.0, 126.9, 126.9, 122.1, 121.0, 115.8 ppm.

**HRMS (ESI)** *m*/*z* calculated for C_45_H_31_N_4_O [M+H]^+^, 643.2492; found, 643.2495. *m*/*z* C_45_H_32_N_4_O [M+2H]^2+^, 322.1283; found, 322.1289.

**UV–Vis** [DCM, λ_max_ (logε), C = 5.3 × 10^−5^ M]: 418 (4.89), 515 (3.53), 548 (3.06), 589 (3.02), 647 (2.85).

**FTIR** (solid,$\;\bar{\nu \;}\left({\mathrm{cm}}^{-1}\right)$) = 1655 (CO)

## 4. Conclusions

We prepared a set of both previously known and unknown meso-substituted porphyrin derivatives, and we conducted a preliminary study of their electrochemical and photophysical properties. Taking meso-tetraphenylporphyrin **TPPH_2_** as the reference compound, we found that while the estimated singlet excited-state energies are essentially unaffected by the introduction of substituents, the redox potentials (both in the ground and in the singlet excited state) strongly depend on the electron-withdrawing or electron-donating nature of the substituents, as well as on their position. Thus, the presence of groups with electron-withdrawing resonance effects (such as formyl or benzoyl) results in an enhancement of the facility of reduction of the photocatalyst, both in the ground and in the excited state. Moreover, this effect increases with the proximity of the electron-accepting substituent to the porphine core, reaching a maximum when a benzoyl substituent is introduced at a meso position. In conclusion, we have shown that the redox properties of meso-substituted porphyrins can be easily tuned toward a reductive quenching pathway in a photoredox catalytic cycle. The study of both the photosensitizing ability (olefin isomerization, photocatalyzed HAT) and the photoredox catalytic activity of the newly synthesized porphyrins is underway in our laboratories, and the results will be reported in due course.

## Figures and Tables

**Table 1:** Ground-state half-wave potentials of porphyrins **TPPH_2_** and **1**–**9** in dichloromethane.

Entry	Porphyrin	Experimental Ground-State Redox Half-Wave Potentials (V vs. SCE)			
		[PC^−^/PC^2−^]	[PC/PC^−^]	[PC^+^/PC]	[PC^2+^/PC^+^]
1	**TPPH_2_ ^a^**	−1.63	−1.21	+1.09	+1.28
2	**1 ^b^**	−1.66 ^c^	−1.21 ^d^/−1.06 ^e^	+1.10 ^f^	+1.20 ^f^
3	**2**	−1.56	−1.22	+1.00 ^f,g^/+1.10 ^f^	+1.70 ^f^
4	**3**	−1.49	−1.14	+1.05 ^f,h^/+1.15	+1.60 ^f^
5	**4**	−1.23	−0.95	+1.11	+1.45
6	**5**	−1.43	−1.07	+1.11	+1.48
7	**6**	−1.37	−1.10	+1.24	+1.56
8	**7**	−1.28	−1.00	+1.22	+1.43
9	**8**	−1.31	−1.09	+1.23	+1.60
10	**9**	−1.17	−0.97	+1.32	+1.62

^a^ See ref. [15] for the literature values in DMSO. ^b^ See ref. [26] for the literature values. ^c^ Process overlapped with reduction of the NO_2_Ph anion. ^d^ Reduction of the porphyrin ring. ^e^ Reduction of the neutral NO_2_Ph group. ^f^ Irreversible. ^g^ Oxidation of the phenylamino group. ^h^ Oxidation of the 4′-pyridyl group.

**Table 2:** Ground- and (singlet) excited-state redox potentials of porphyrins **TPPH_2_** and **1**–**9**.

Entry	Porphyrin	Ground-State Redox Potentials (V vs. SCE)		(Singlet) Excited-State Redox Potentials (V vs. SCE)	
		[PC/PC^−^]	[PC^+^/PC]	[*PC/PC^−^]	[PC^+^/*PC]
1	**TPPH_2_^a^**	−1.21	+1.09	+0.70	−0.82
2	**1**	−1.06	+1.10	+0.85	−0.81
3	**2**	−1.22	+1.00	+0.69	−0.91
4	**3**	−1.14	+1.05	+0.77	−0.86
5	**4**	−0.95	+1.11	+0.91	−0.80
6	**5**	−1.07	+1.11	+0.93	−0.90
7	**6**	−1.10	+1.24	+0.81	−0.71
8	**7**	−1.00	+1.22	+0.89	−0.67
9	**8**	−1.09	+1.23	+0.91	−0.77
10	**9**	−0.97	+1.32	+0.94	−0.59

^a^ See ref. [15] for the literature values in DMSO.

**Table 3:** Selected photophysical data for porphyrins **6**–**9** in dichloromethane.

Porphyrin	Less Energetic Absorption Q Band (nm)	More Energetic Emission Q(0,0) Band (nm)	Normalized Intersection Wavelength (nm)	Relative Fluorescence Quantum Yield (ϕ_f_) ^a^	Estimated Singlet Excited-State Energies (V)
**6**	645	651	648	0.09	1.91
**7**	651	662	657	0.09	1.89
**8**	587	650	618	0.002	2.00
**9**	647	650	648	0.08	1.91

^a^ **H_2_TPP** [(ϕ_f_) = 0.11] was used as the reference compound.

## Data Availability

No new data were created in addition to those reported here and in the Supplementary Materials.

## Supplementary Materials

The following supporting information can be downloaded at: https://www.mdpi.com/article/10.3390/molecules29153689/s1: ^1^H and ^13^C NMR spectra for porphyrins **6**, **7**, **9**, **11**, and **12** (ESI2-ESI6); cyclic voltammetries for compounds **TPPH_2_**, **1**–**9** (ESI7-ESI10); UV–Vis and fluorescence spectra for compounds **1**–**9** (ESI11-ESI19).
