# Supplementary material for: Substituent Effects in the Photophysical and Electrochemical Properties of Meso-Tetraphenylporphyrin Derivatives
Source: Molecules. 2024 Aug 4;29(15):3689. doi: 10.3390/molecules29153689 (PMC11314014; doi:10.3390/molecules29153689)

Molecules
Supplementary Material:

Electronic Supporting Information for

Substituent Effects in the Photophysical and Electrochemical Properties of meso-Tetraphenylporphyrin Derivatives

Alexandra Cruz Millheim, Enric Ponzano and Albert Moyano*

Section of Organic Chemistry, Department of Inorganic and Organic Chemistry, Faculty of Chemistry, University of Barcelona, C. de Martí i Franquès 1-11, 08028 Barcelona, Spain

SUMMARY

- ^{1}H and ^{13}C NMR spectra for porphyrins **6**, **7**, **9**, **11** and **12** (ESI2-ESI6)
- Cyclic voltammetries for compounds **TPPH$_2$**, **1-9** (ESI7-ESI10)
- UV-Vis and fluorescence spectra for porphyrins **1-9** (ESI11-ESI19)

^{1}H-NMR spectrum (400 MHz, $CDCl_3$) of compound **6**

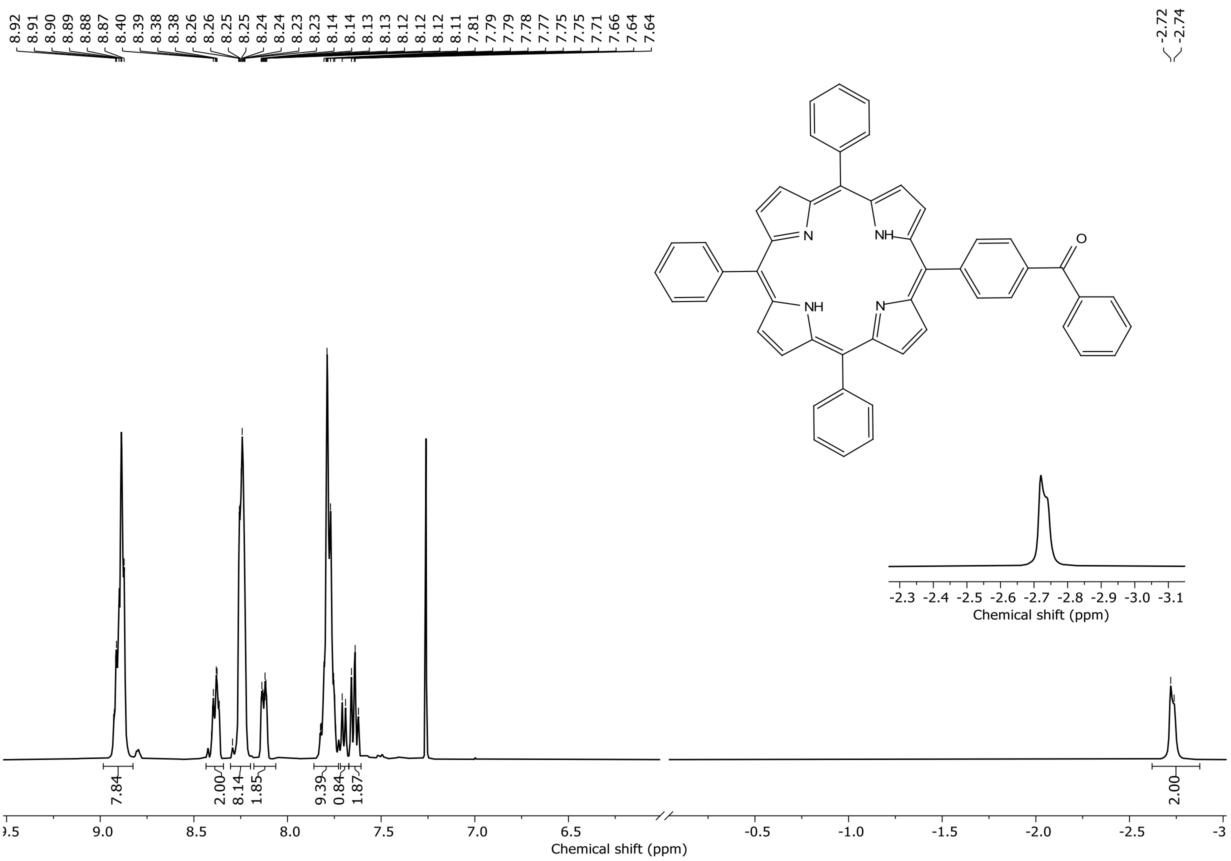

^{13}C-NMR spectrum (101 MHz, $CDCl_3$) of compound **6**

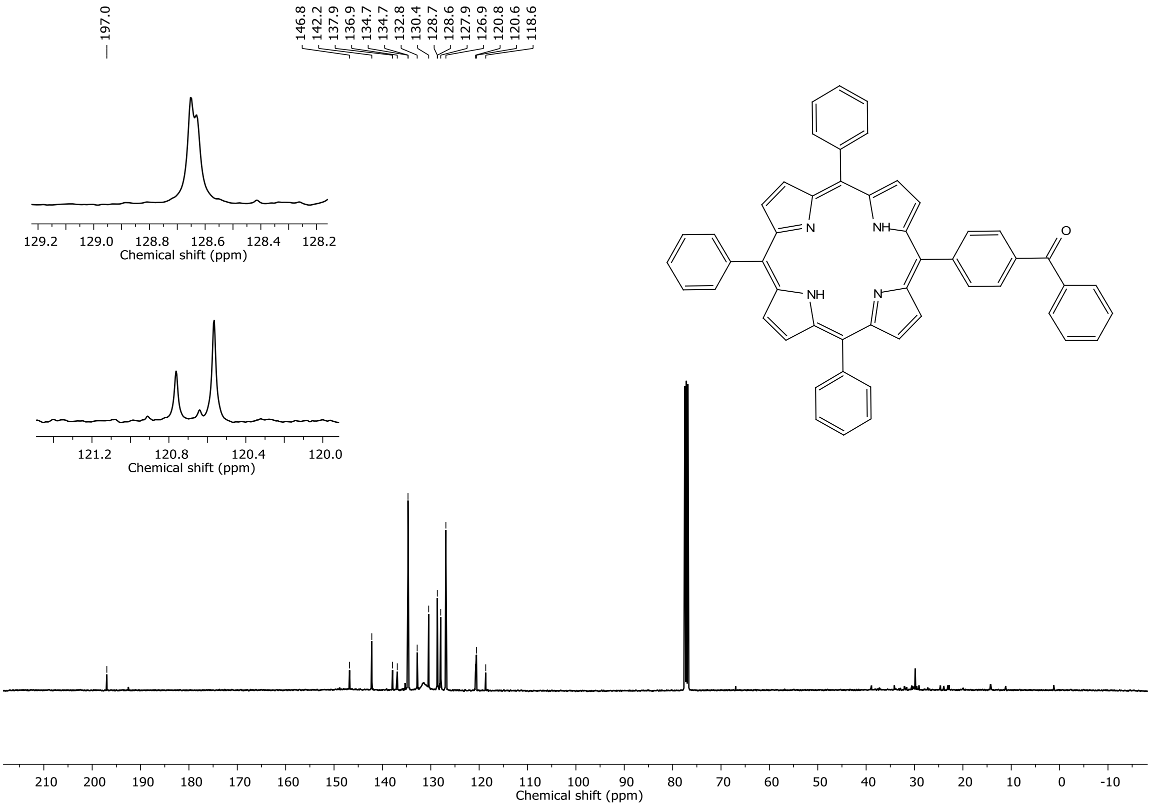

^{1}H-NMR spectrum (400 MHz, $CDCl_3$) of compound **7**

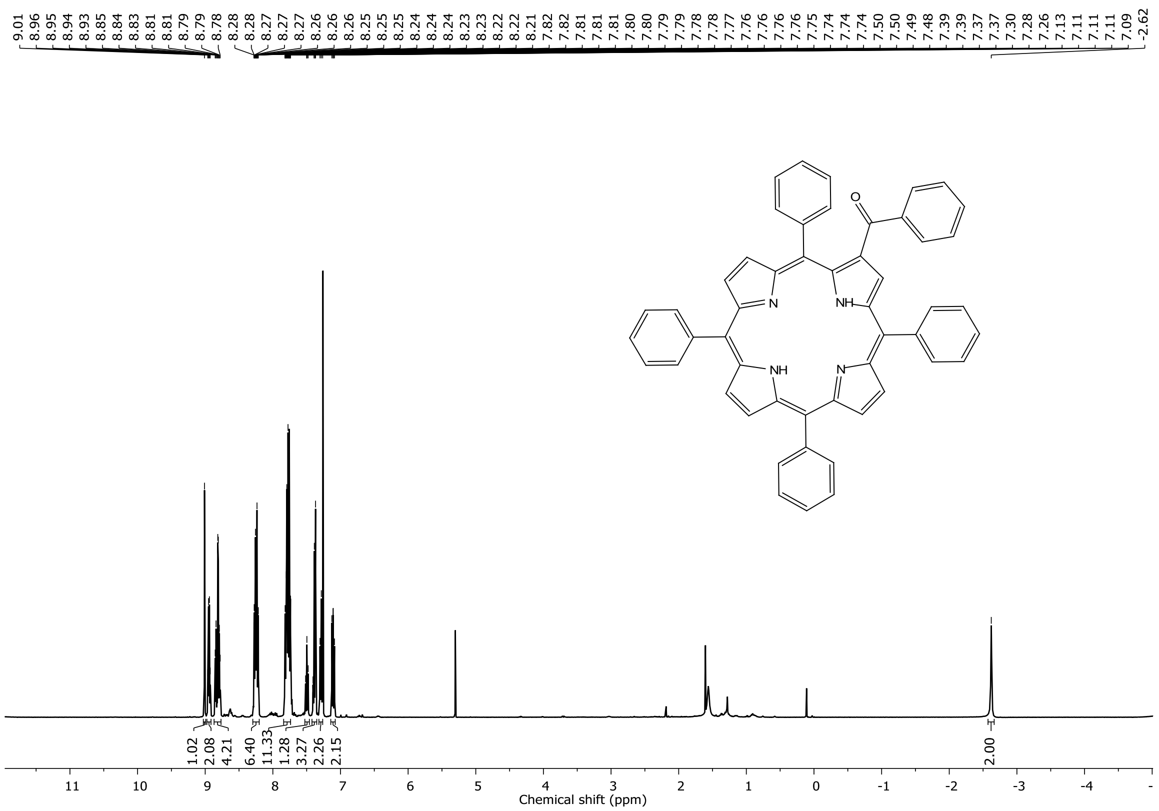

^{13}C-NMR spectrum (101 MHz, $CDCl_3$) of compound **7**

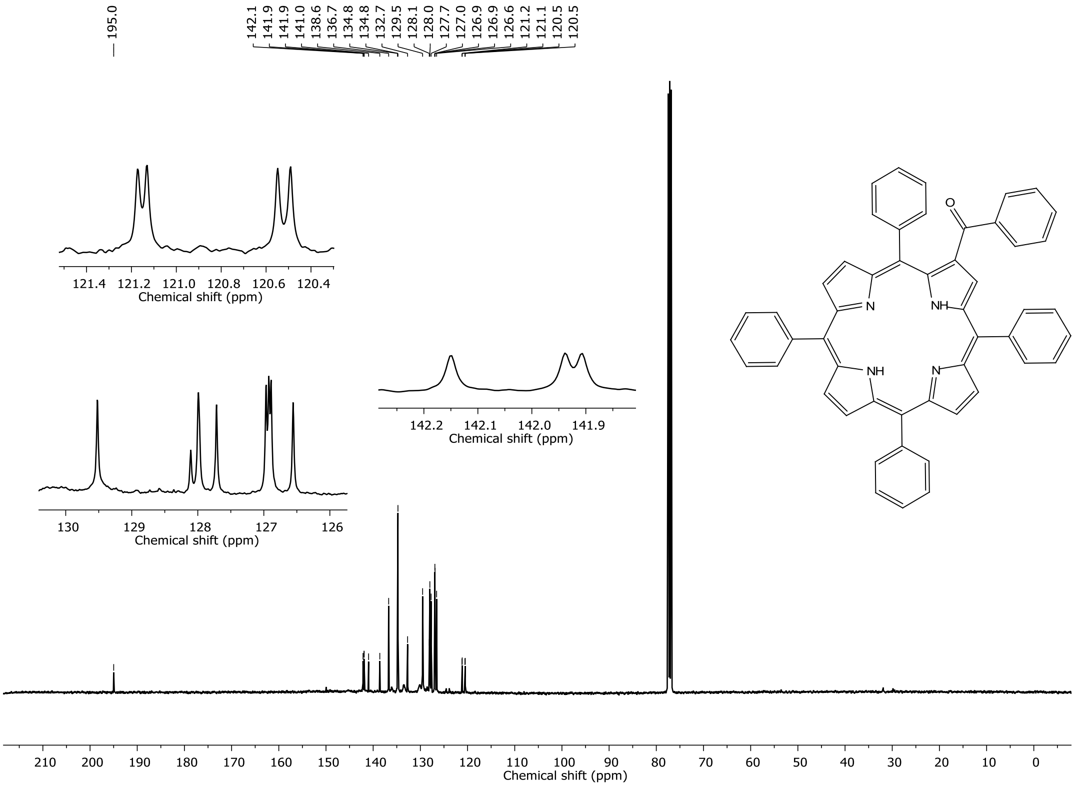

^{1}H-NMR spectrum (400 MHz, $CDCl_3$) of compound **9**

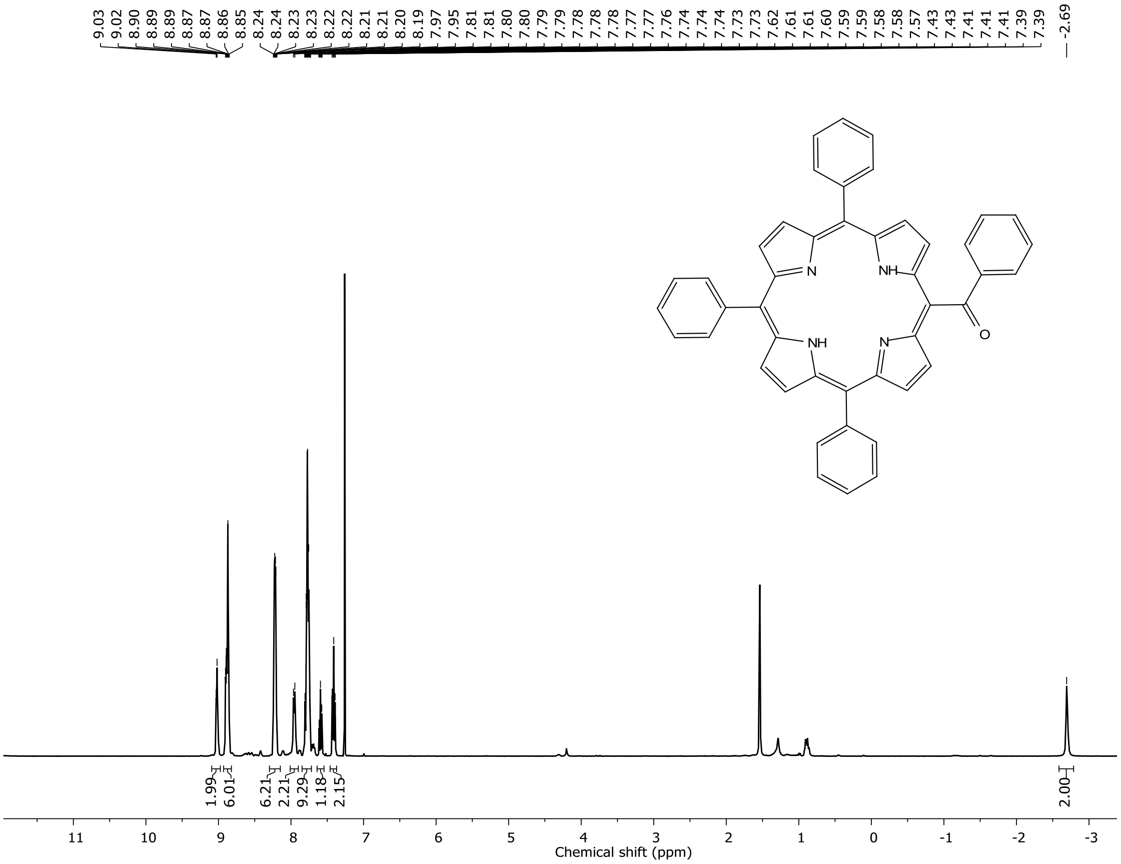

^{13}C-NMR spectrum (101 MHz, $CDCl_3$) of compound **9**

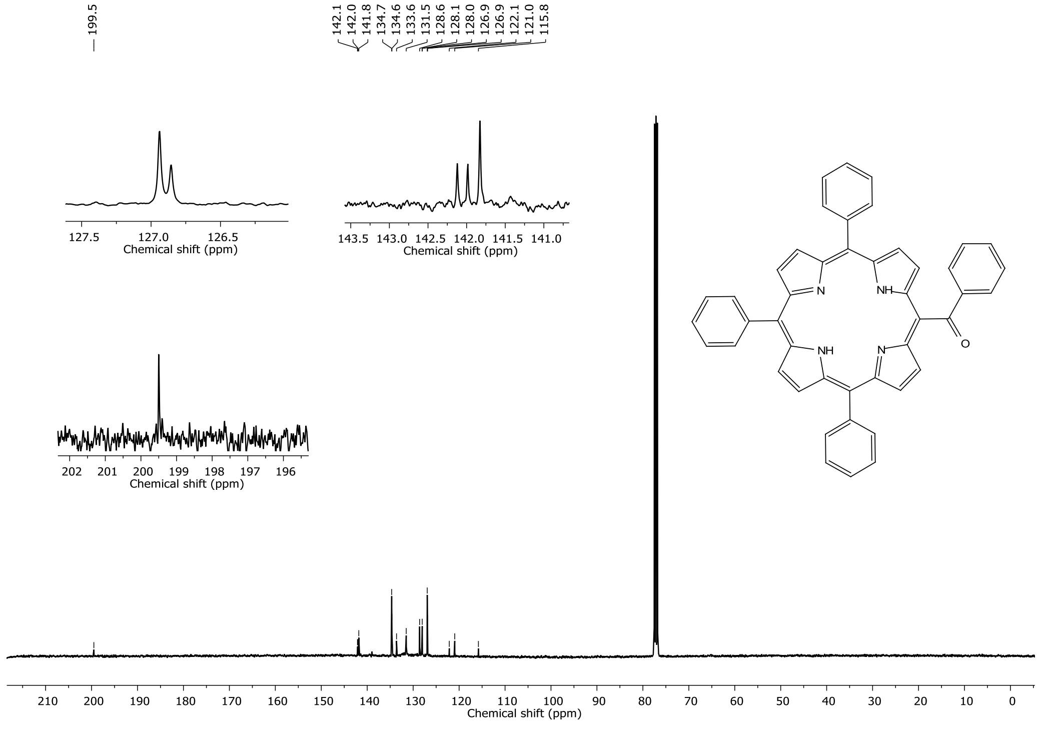

^{1}H-NMR spectrum (400 MHz, $CDCl_3$) of compound **11**

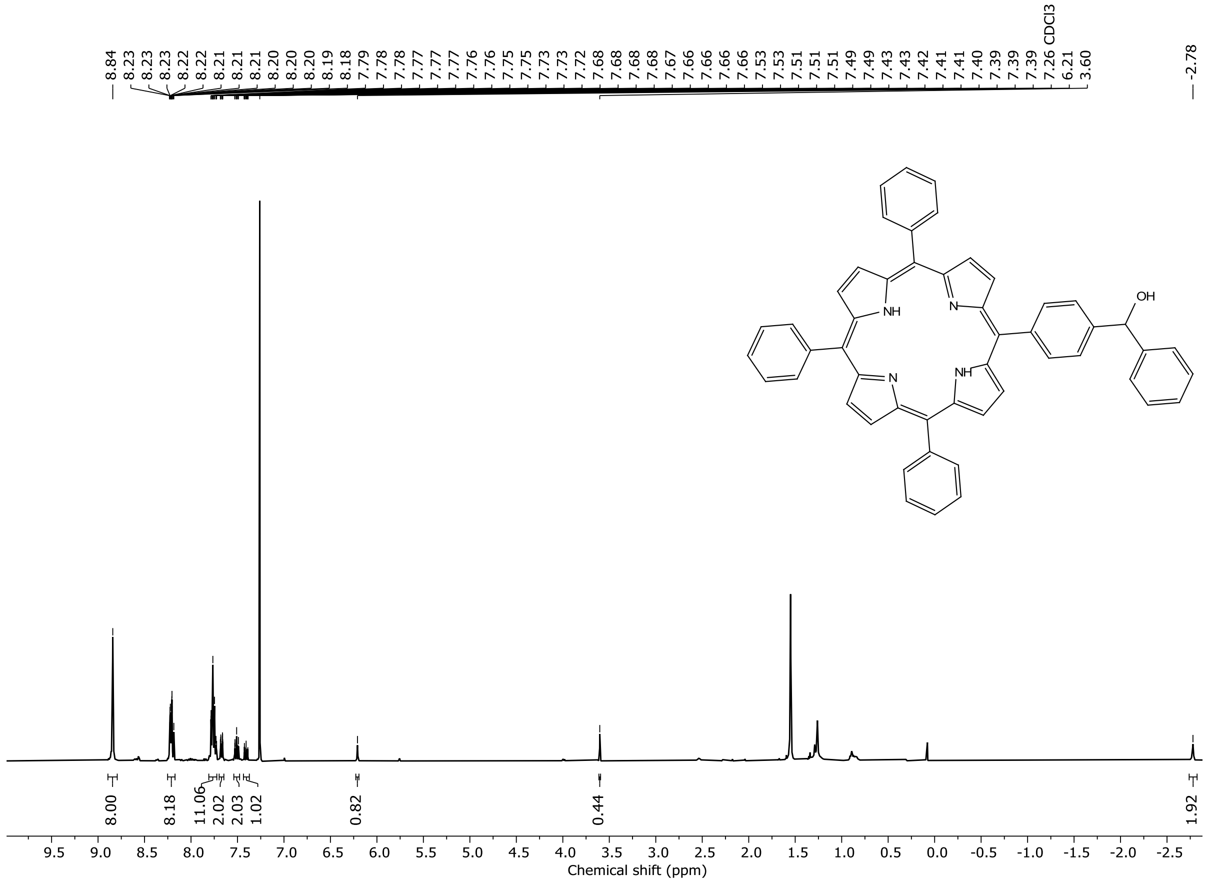

^{13}C-NMR spectrum (101 MHz, $CDCl_3$) of compound **11**

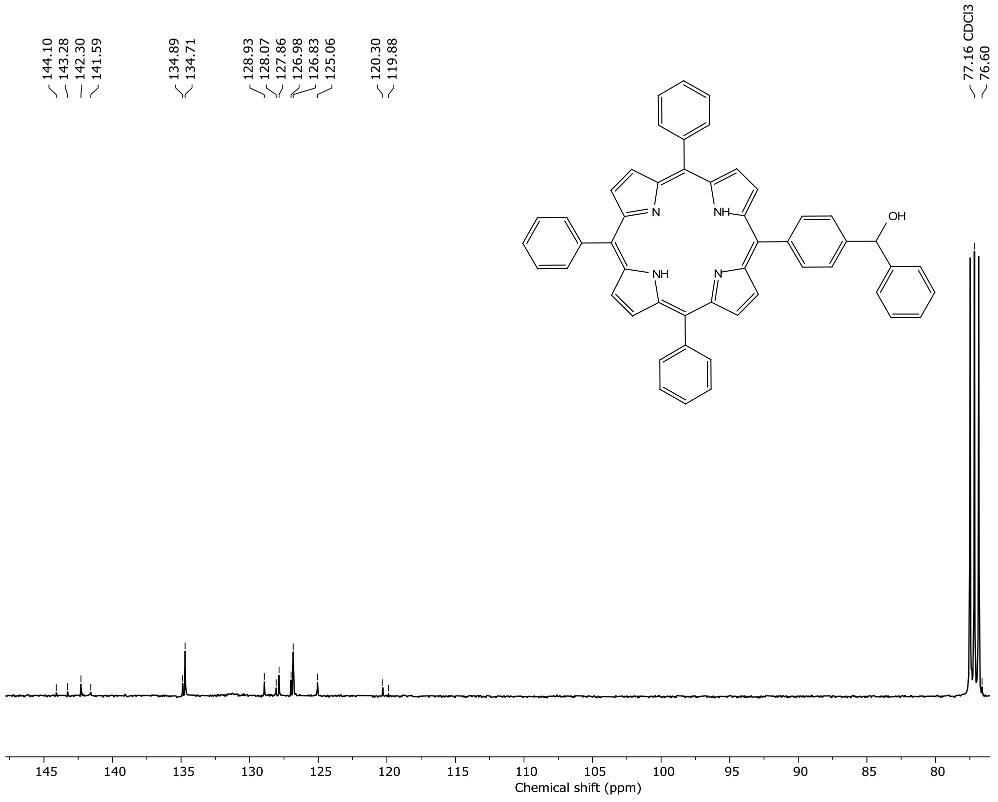

^{1}H-NMR spectrum (400 MHz, $CDCl_3$) of compound **12**

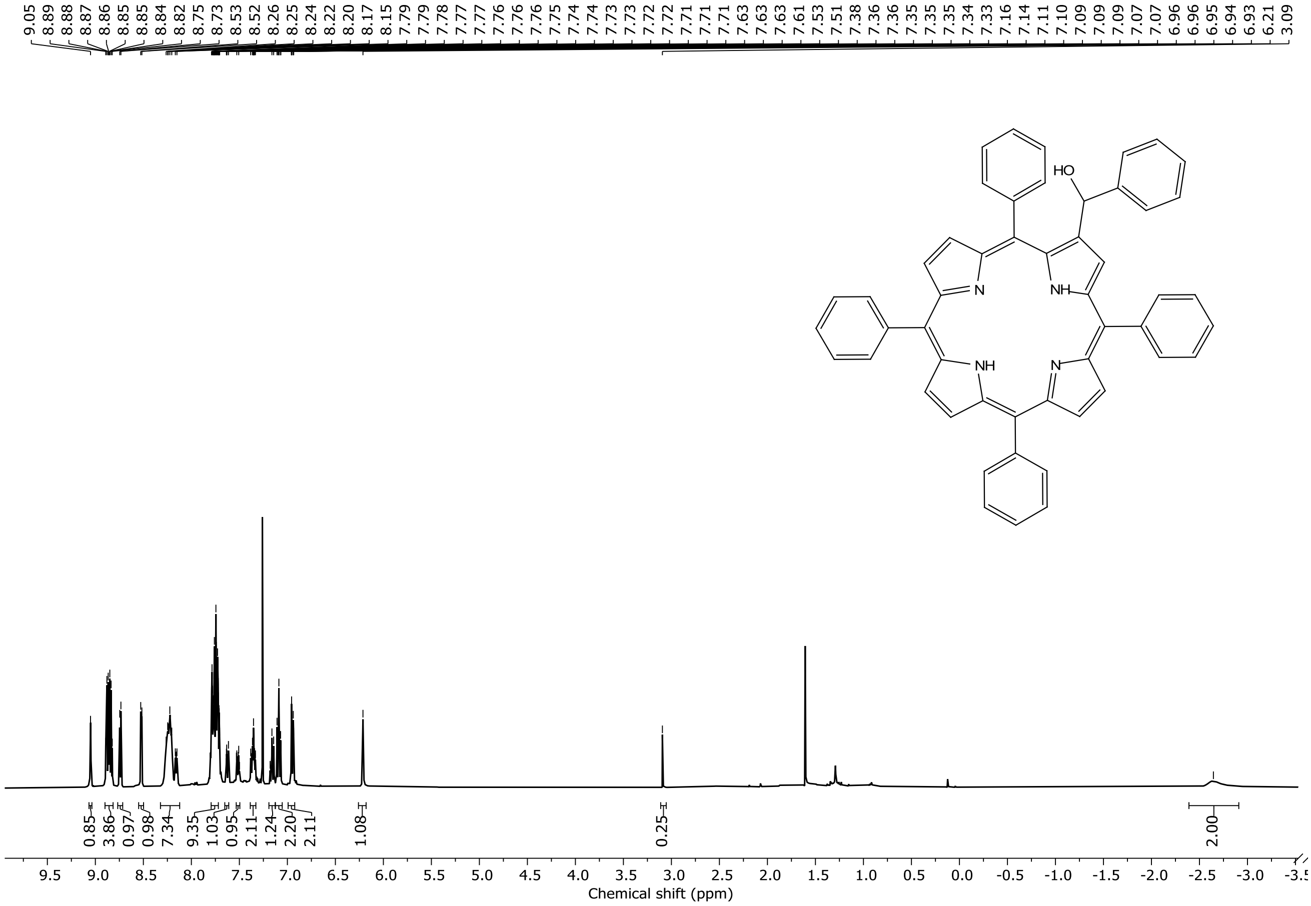

^{13}C-NMR spectrum (101 MHz, $CDCl_3$) of compound **12**

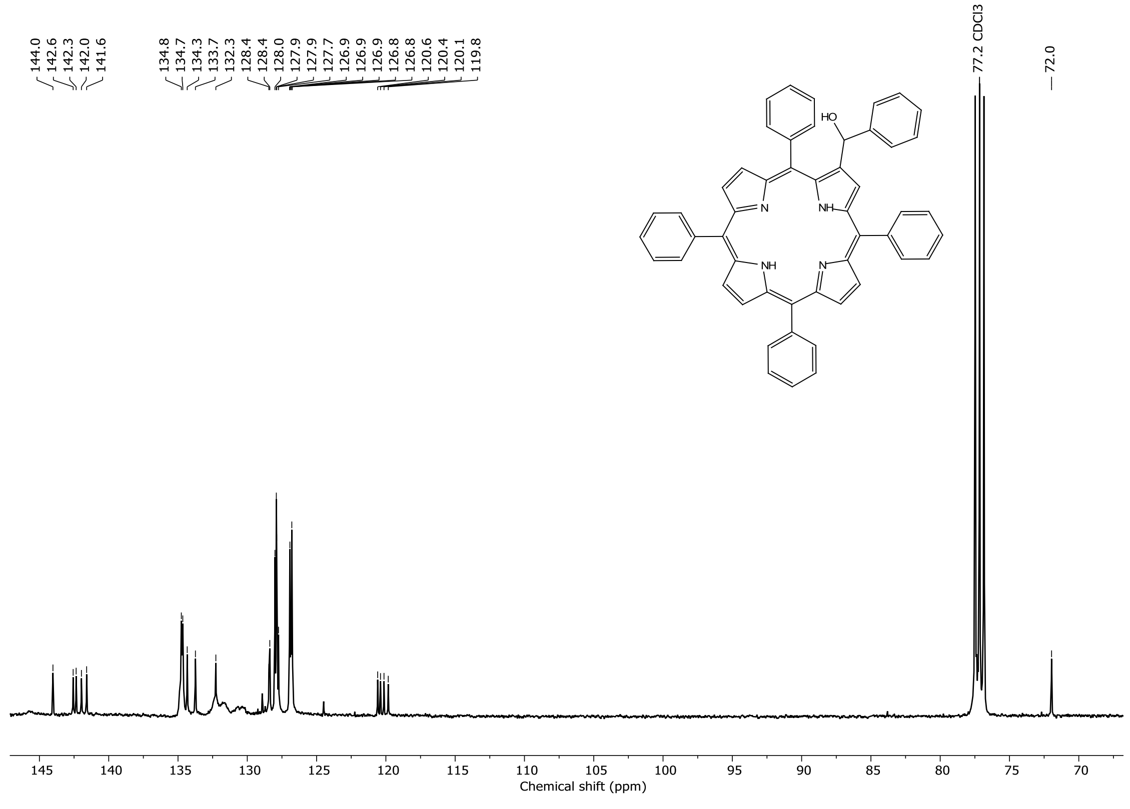

Cyclic voltammogram of **TPPH$_2$** (DCM, Ag/AgCl electrode, TBAP 0,1M).

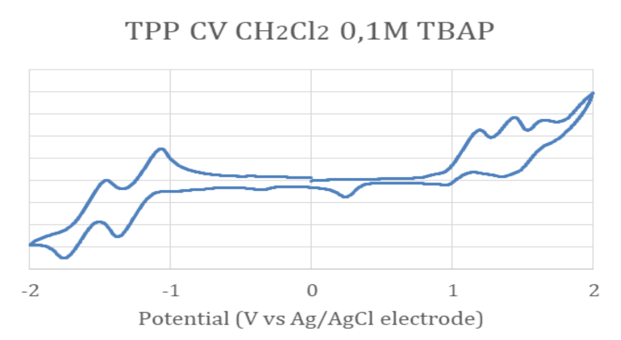

Cyclic voltammogram of **5-(pNO$_2$Ph)TPPH$_2$ 1** (DCM, Ag/AgCl electrode, TBAP 0,1M).

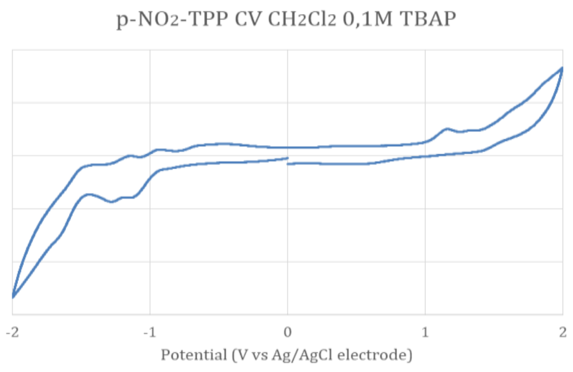

Cyclic voltammogram of **5-(pNH$_2$Ph)TPPH$_2$ 2** (DCM, Ag/AgCl electrode, TBAP 0,1M).

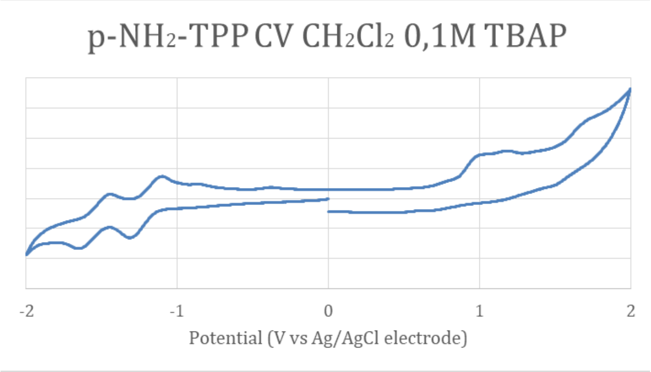

Cyclic voltammogram of **5-(4'-Pyr)TPPH$_2$ 3** (DCM, Ag/AgCl electrode, TBAP 0,1M).

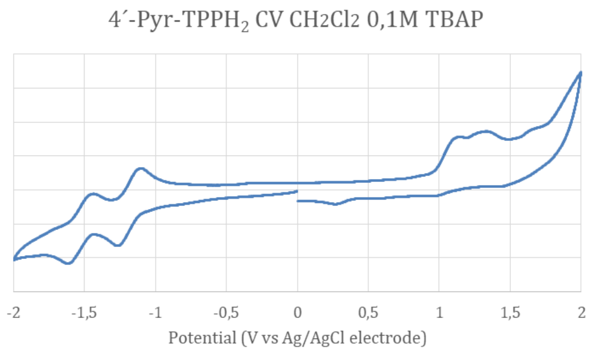

Cyclic voltammogram of **2-(CHO)TPPH$_2$ 4** (DCM, Ag/AgCl electrode, TBAP 0,1M).

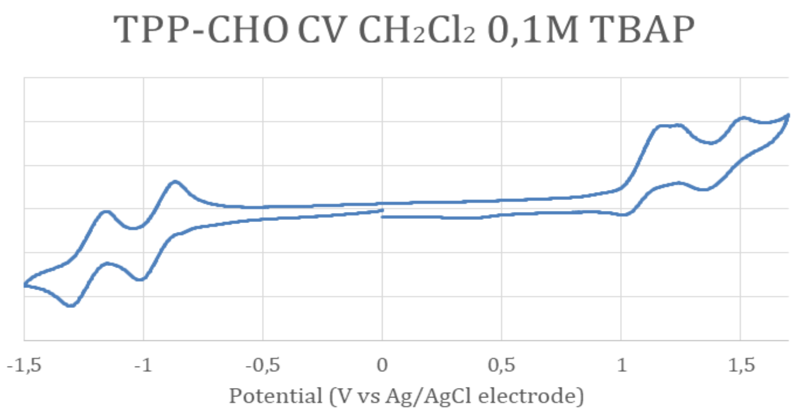

Cyclic voltammogram of **2-(CHO)TPPCu 5** (DCM, Ag/AgCl electrode, TBAP 0,1M).

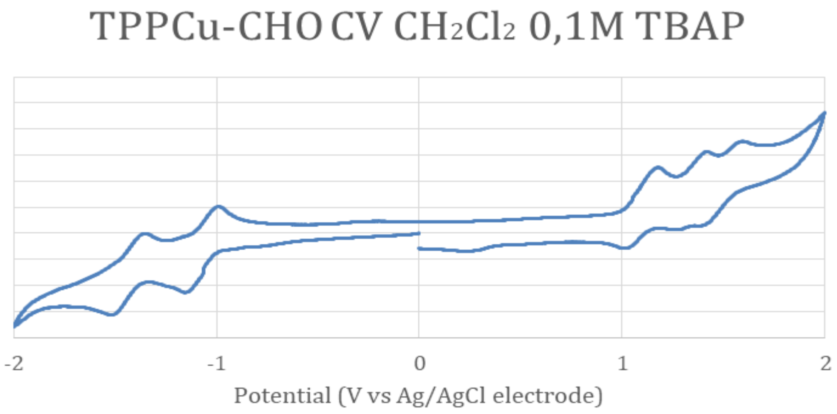

Cyclic voltammogram of **2-(*p*-(PhCO)Ph)TPPH$_2$ 6** (DCM, Ag/AgCl electrode, TBAP 0,1M).

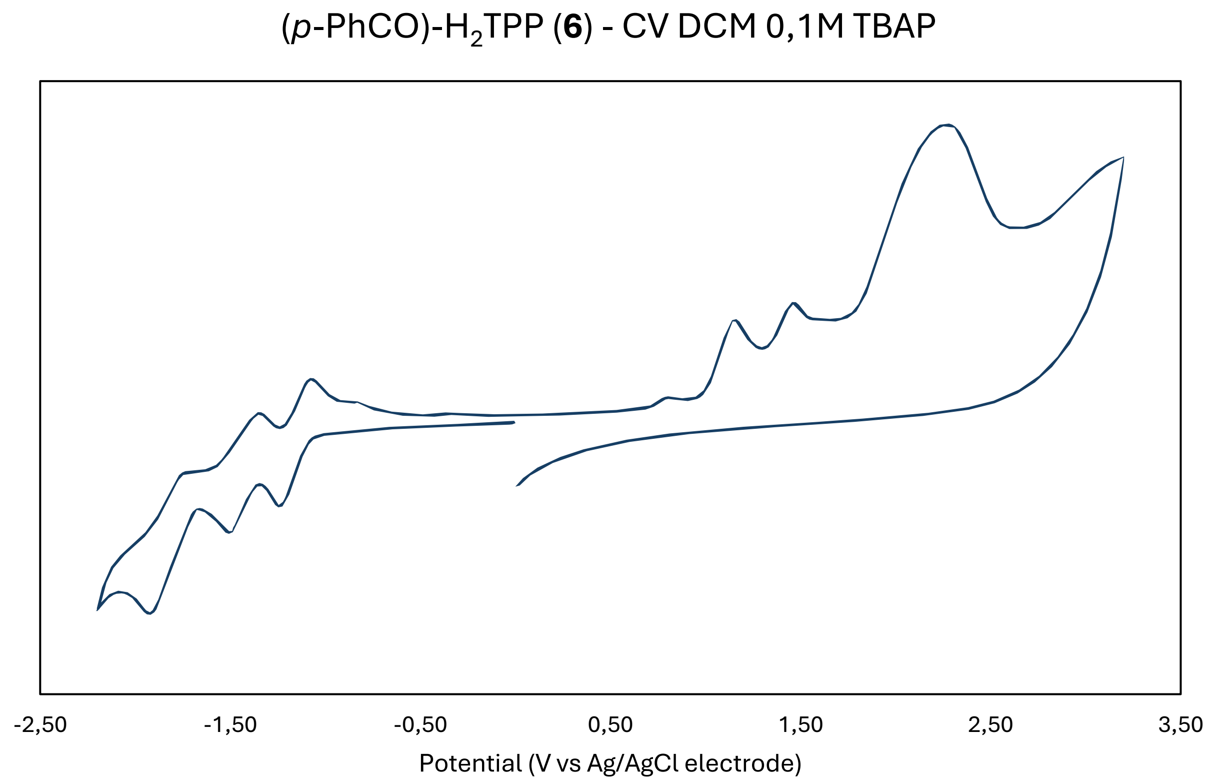

Cyclic voltammogram of **2-(PhCO)TPPH$_2$ 7** (DCM, Ag/AgCl electrode, TBAP 0,1M).

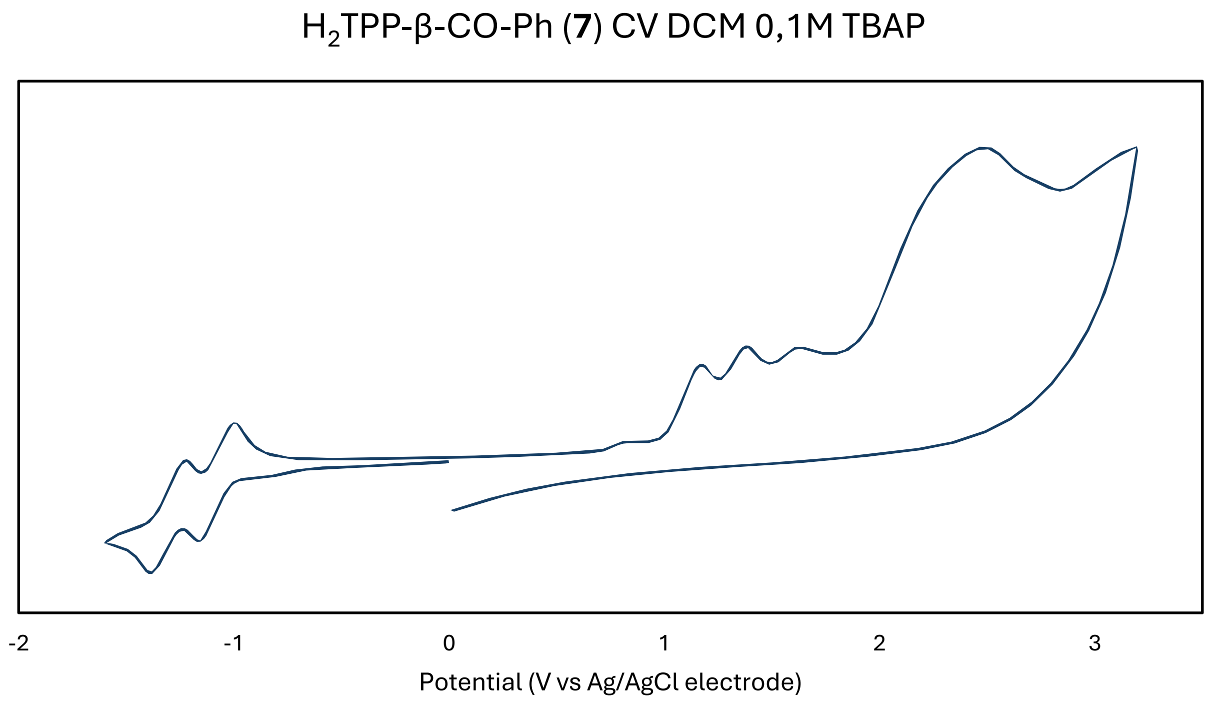

Cyclic voltammogram of **2-(PhCO)TPPCu 8** (DCM, Ag/AgCl electrode, TBAP 0,1M).

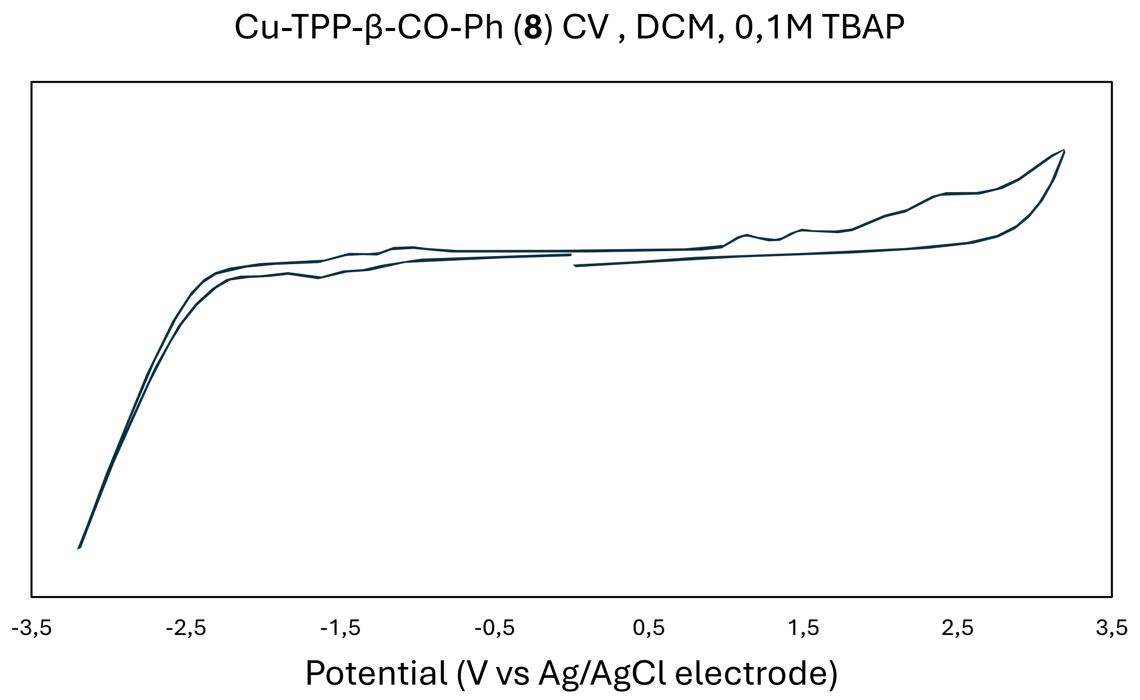

Cyclic voltammogram of **5-(PhCO)TPPH$_2$ 9** (DCM, Ag/AgCl electrode, TBAP 0,1M).

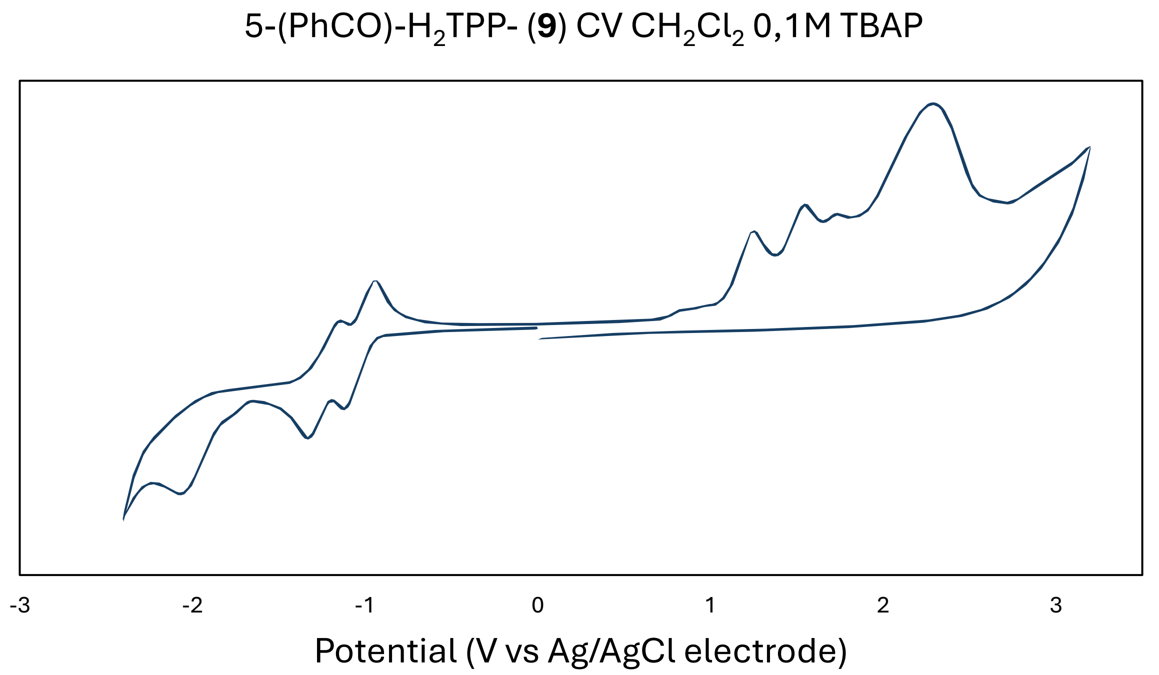

Molecule 1 (5-(4-nitrophenyl)-10,15,20-tetraphenylporphyrin

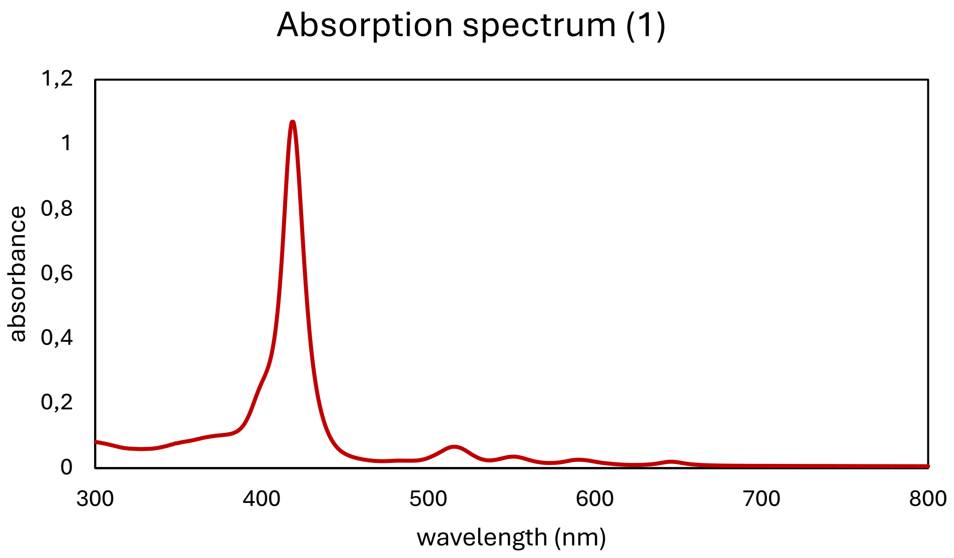

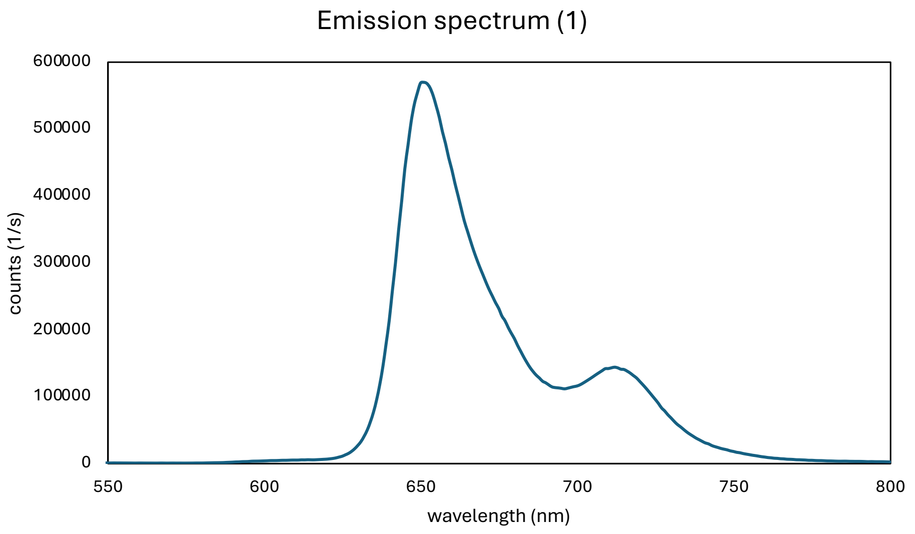

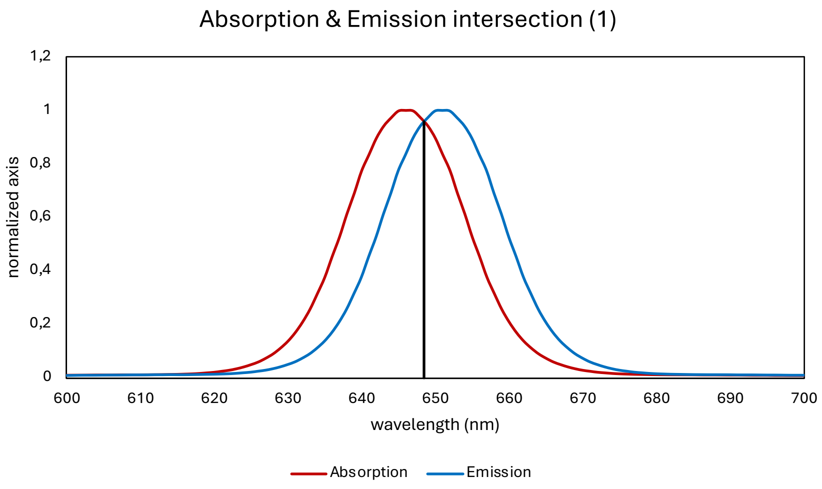

Intersection wavelength: 648 nm

Molecule 2 (5-(4-aminophenyl)-10,15,20-triphenylporphyrin)

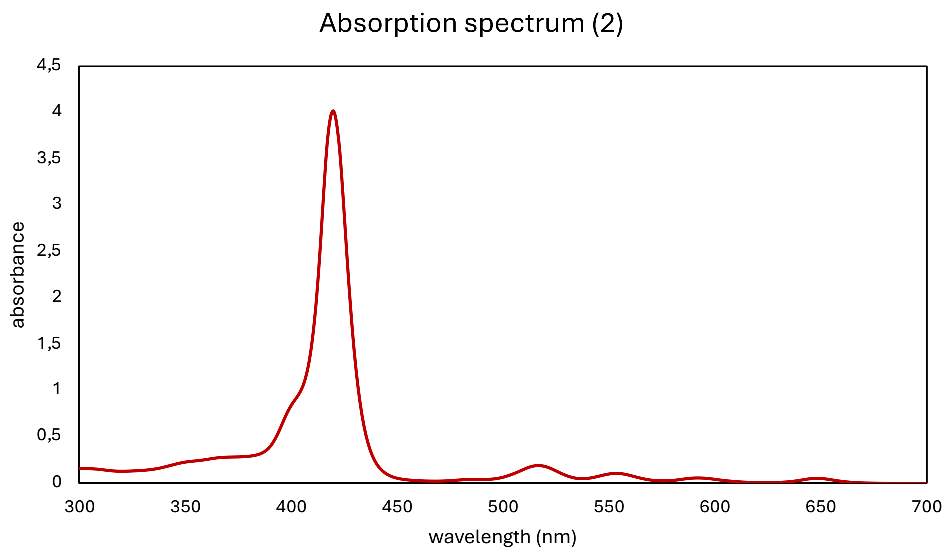

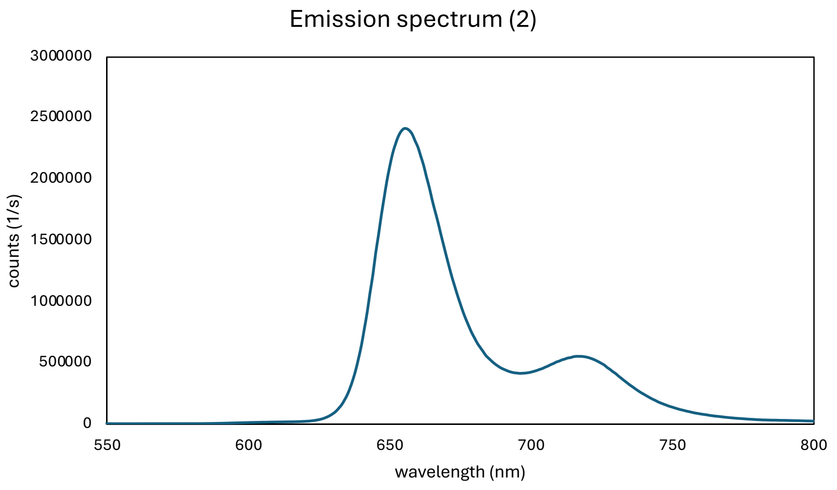

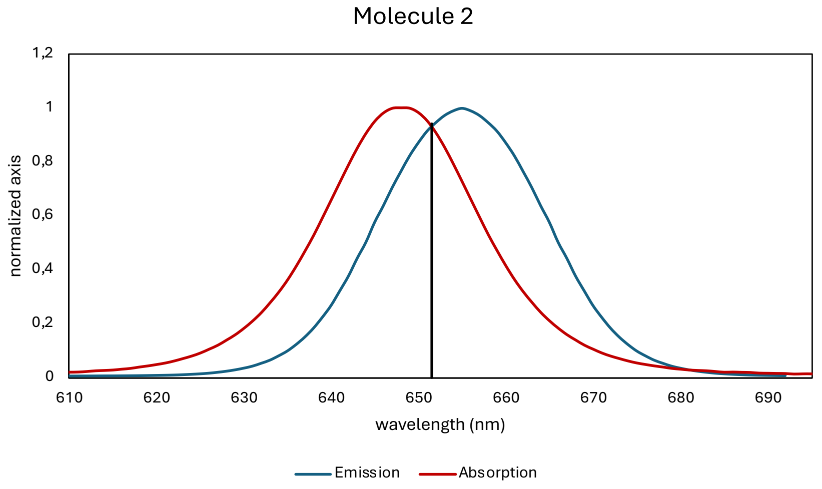

Intersection wavelength: 652 nm

Molecule 3 (5-(4’-piridyl)-10,15,20-triphenylporphyrin)

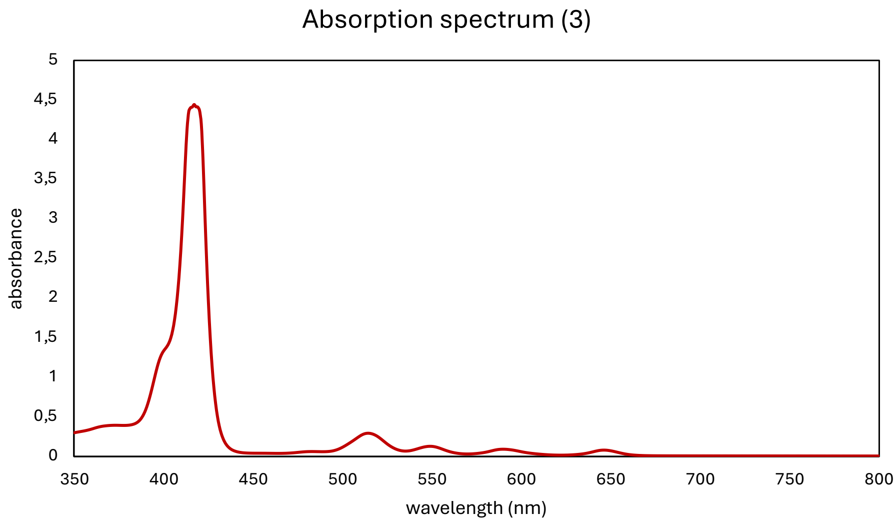

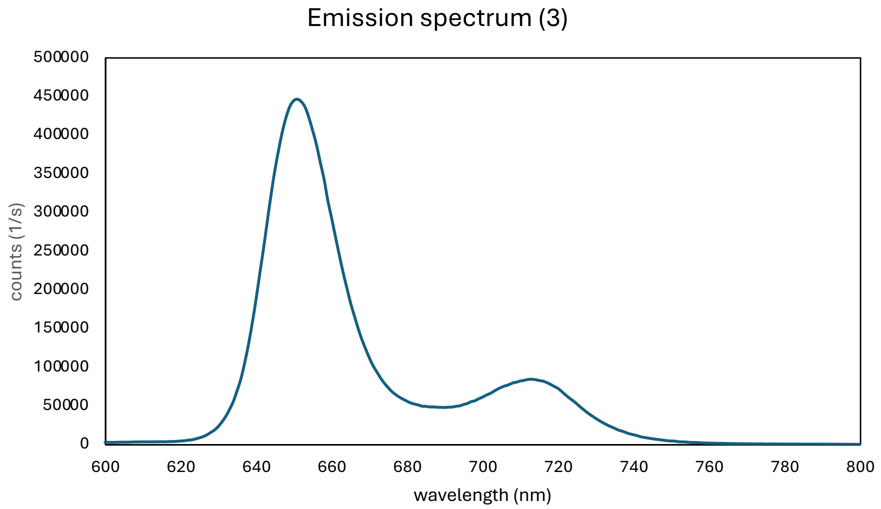

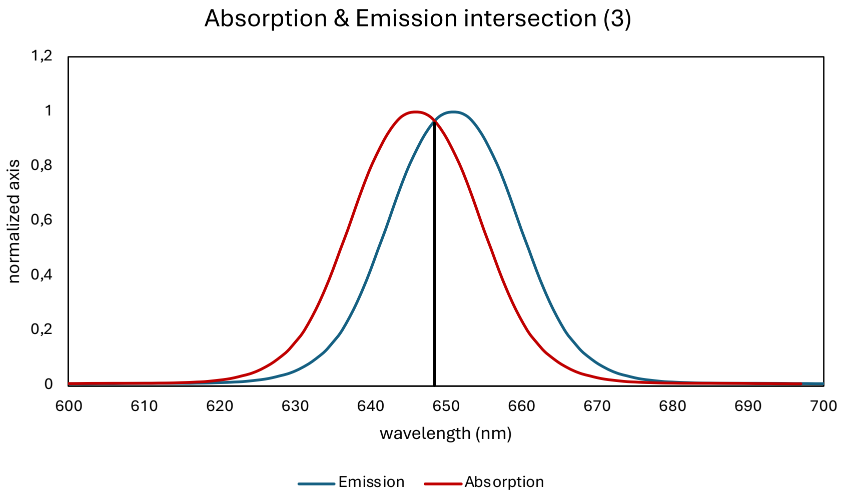

Intersection wavelength: 649 nm

Molecule 4 (7-formyl-5,10,15,20-tetraphenylporphyrin)

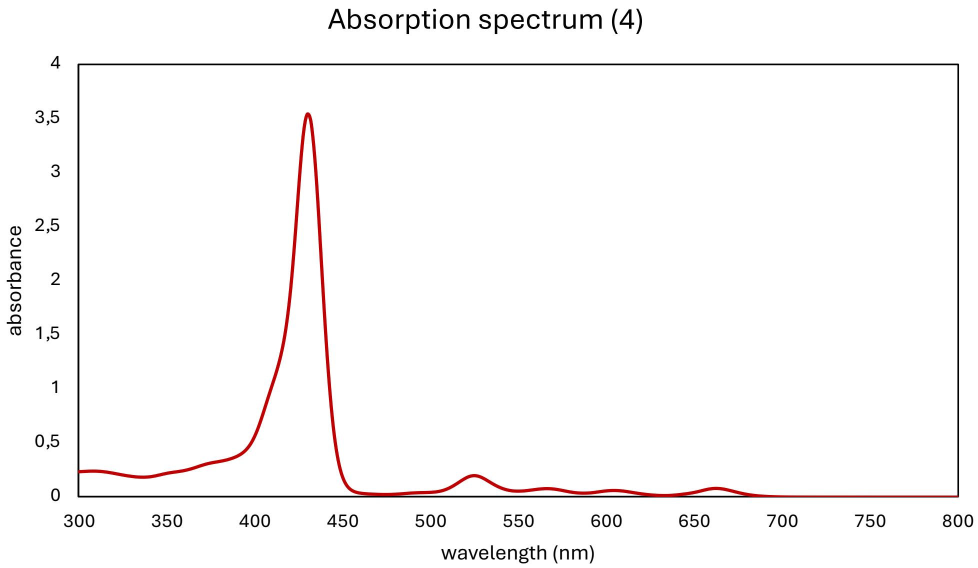

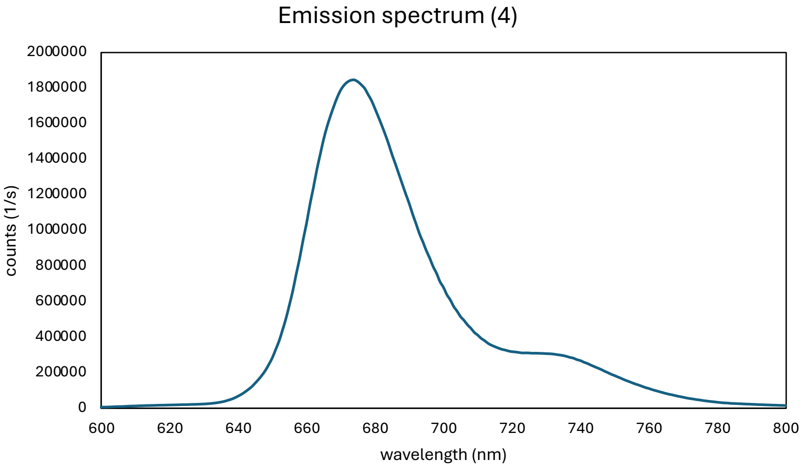

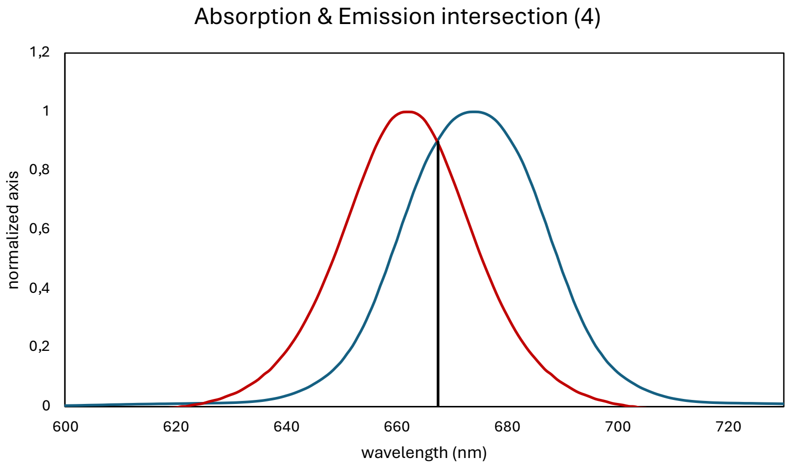

Intersection wavelength: 668 nm

Molecule 5 (7-formyl-5,10,15,20-tetraphenylporphyrin) Cu(II) complex

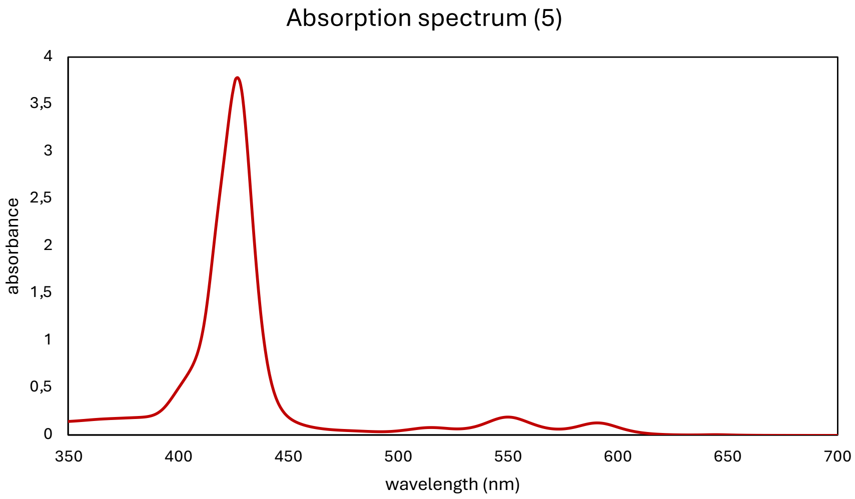

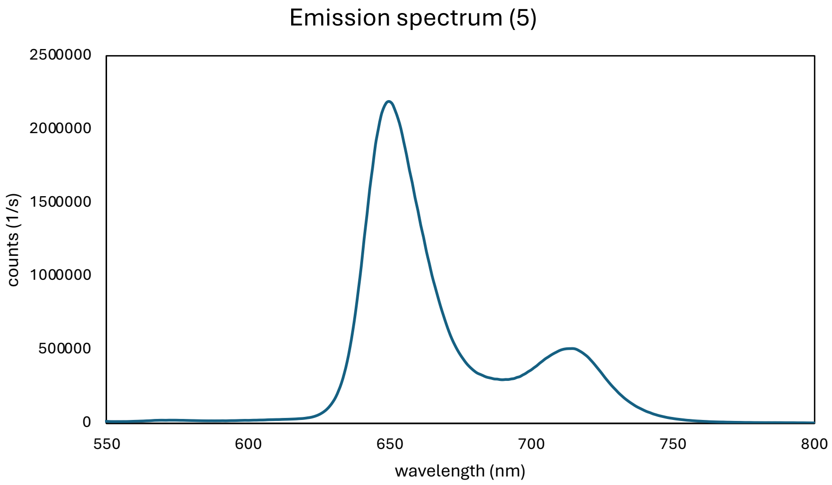

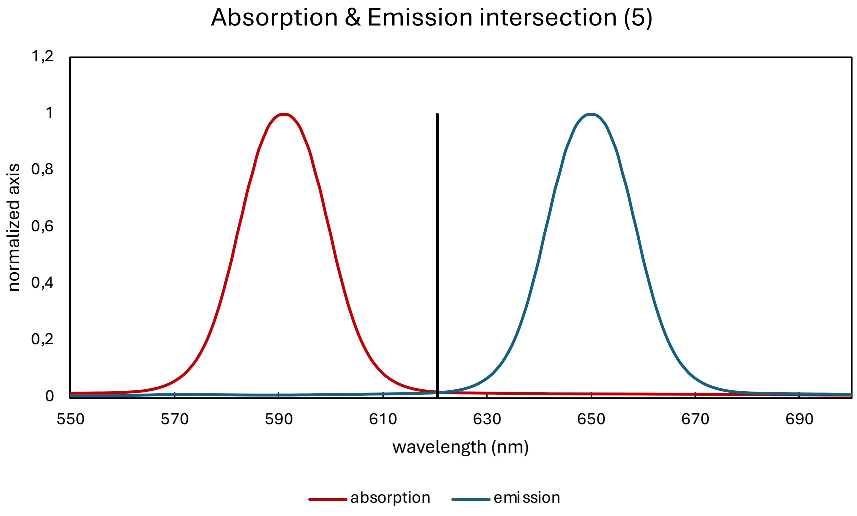

Intersection wavelength: 621 nm

Molecule 6 (5-(4'-Benzoyl)-10,15,20-triphenylporphyrin)

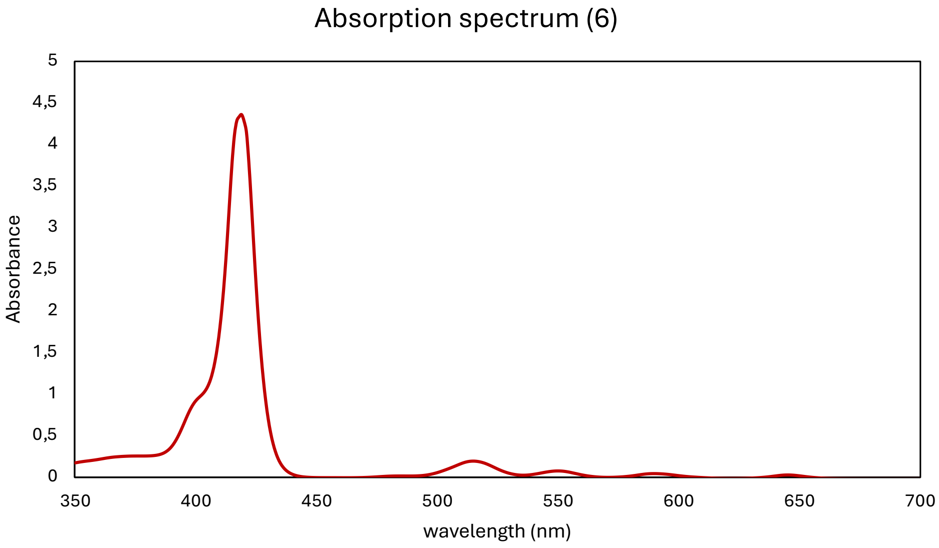

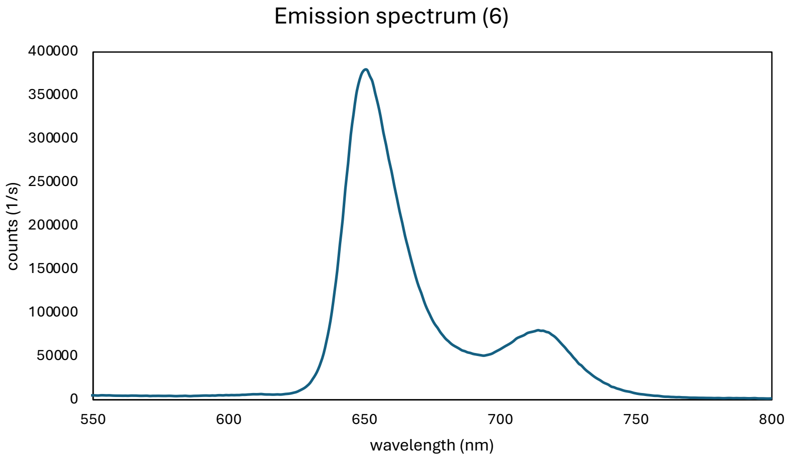

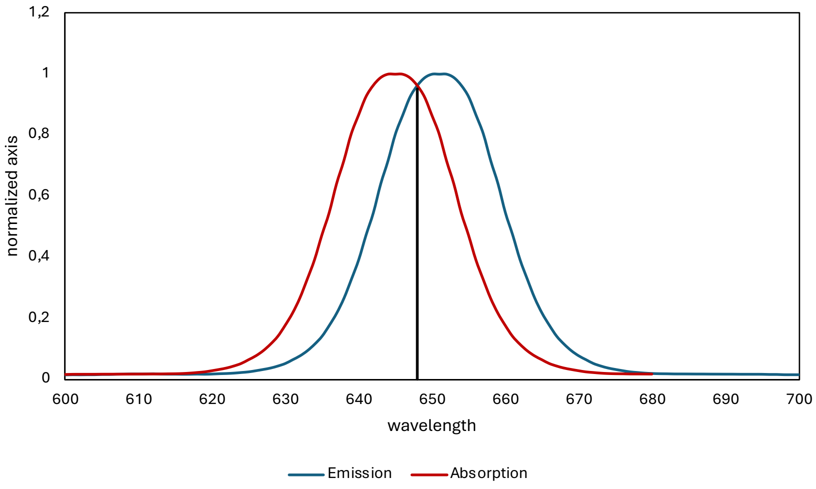

Intersection wavelength: 648 nm

Molecule 7 (Phenyl(5,10,15,20-tetraphenylporphyrin-7-yl)methanone)

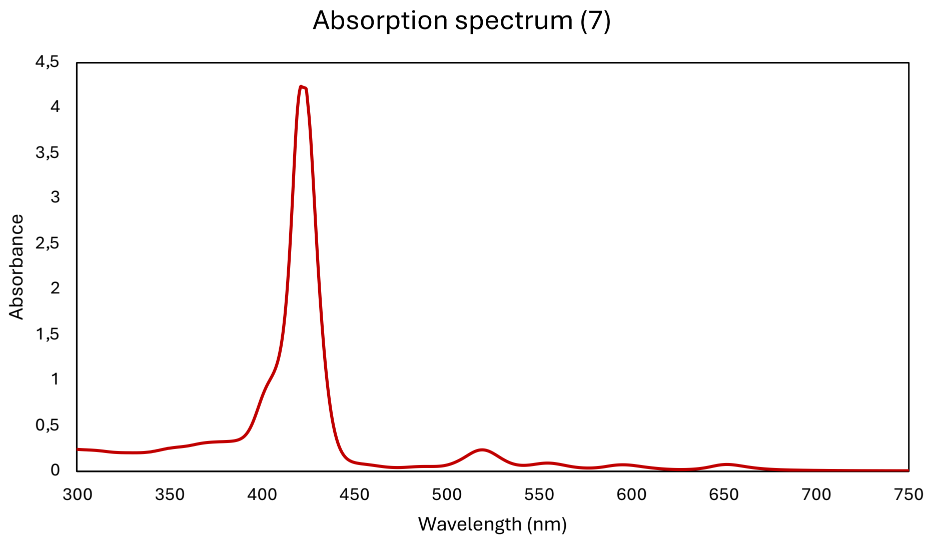

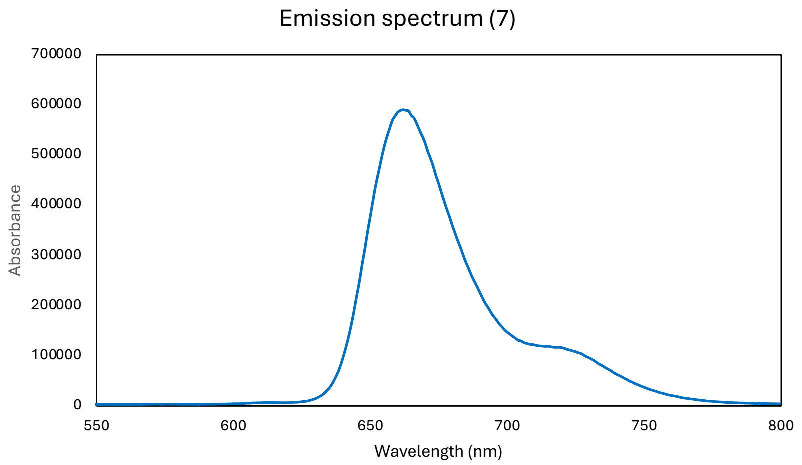

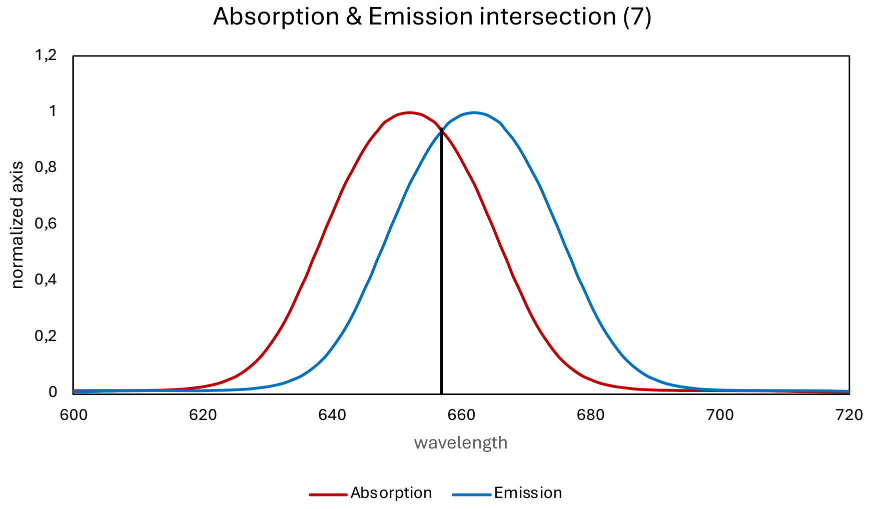

Intersection wavelength: 657 nm

Molecule 8 (Phenyl(5,10,15,20-tetraphenylporphyrin-7-yl)methanone Cu(II) complex)

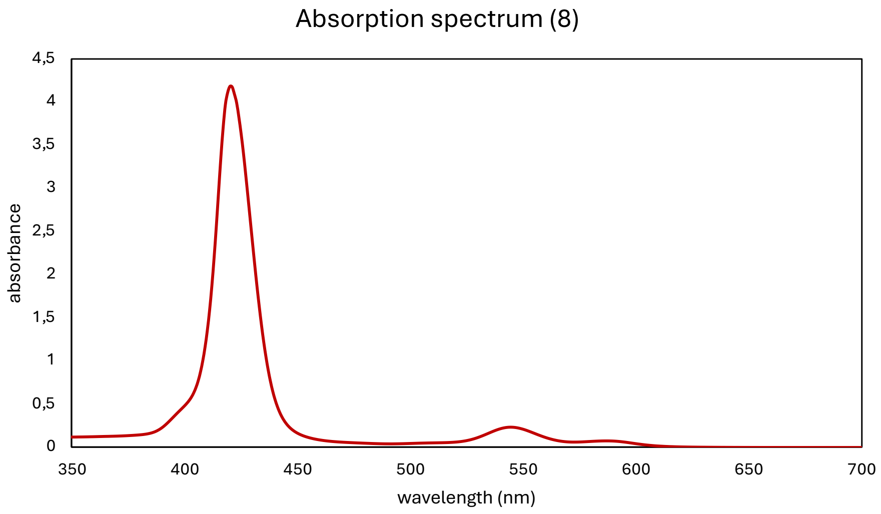

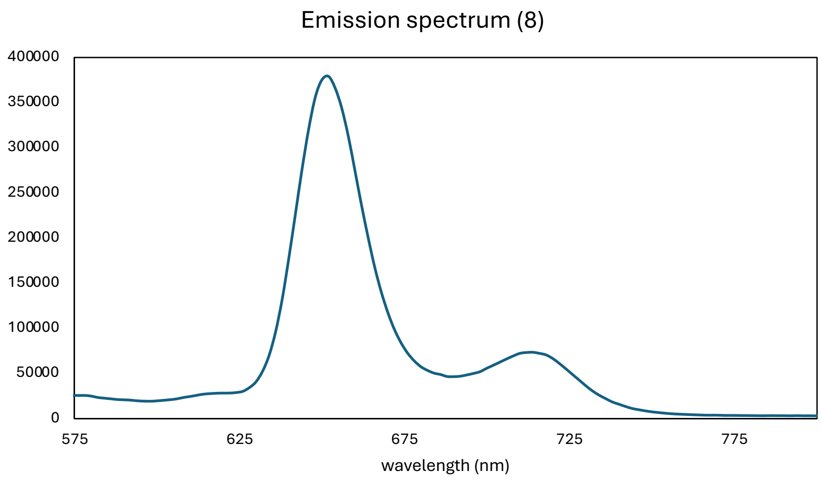

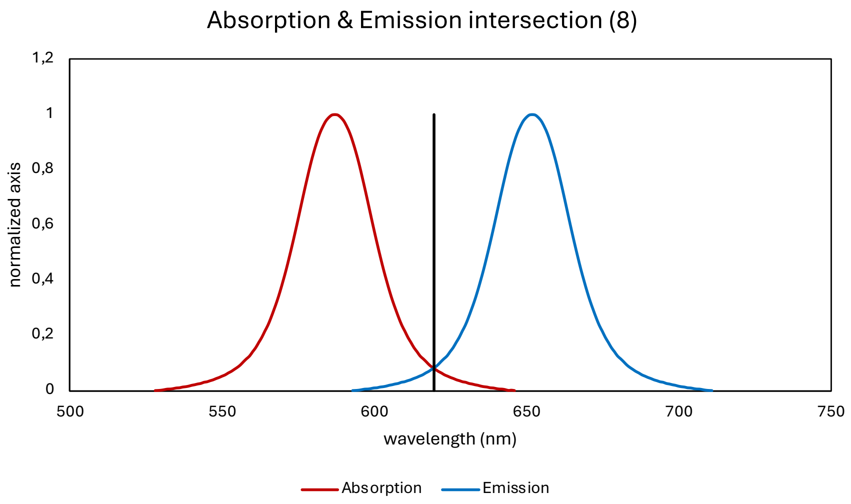

Intersection wavelength: 621 nm

Molecule 9 (5-(Benzoyl)-10,15,20-triphenylporphyrin)

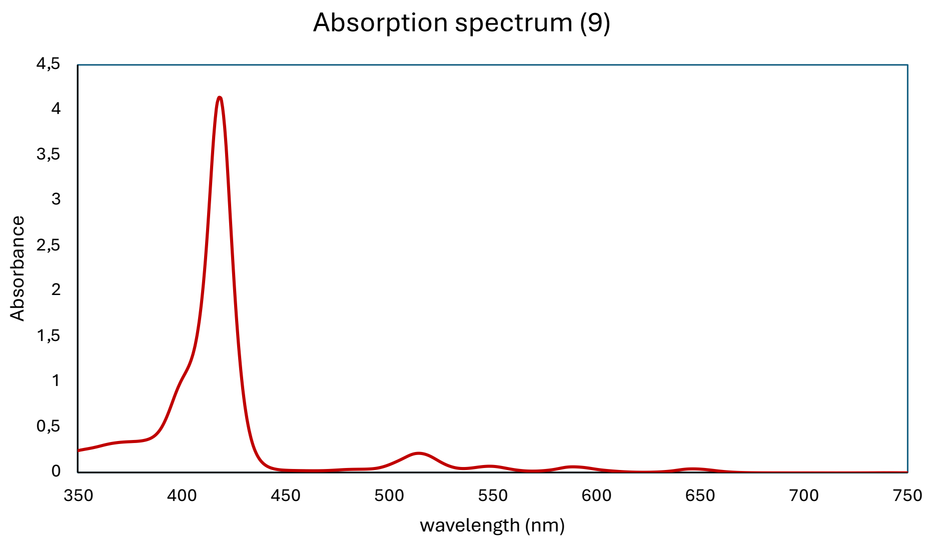

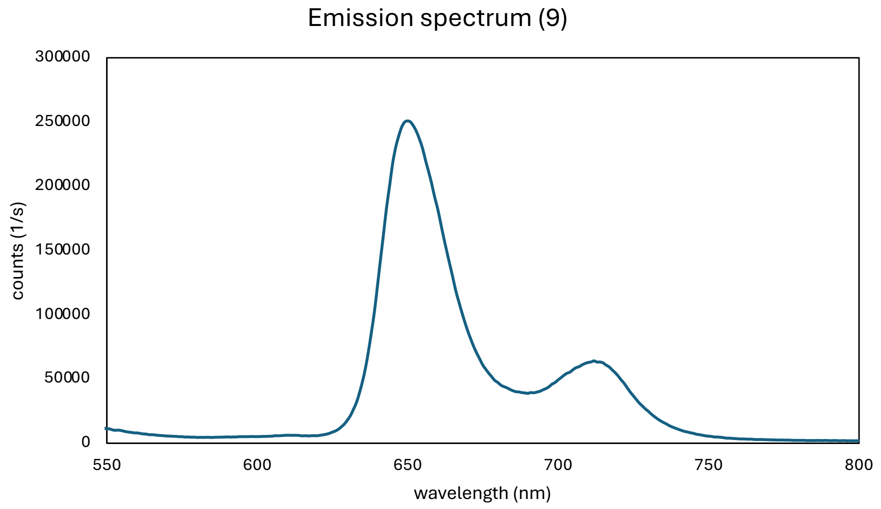

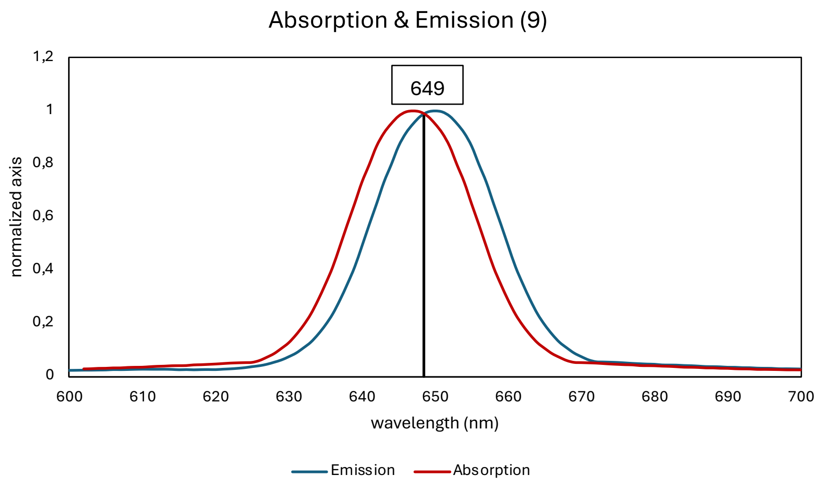

Supplement: Supplementary file 1 [file molecules-29-03689-s001.zip › molecules-3091400-supplementary.pdf]
